# Using Health Information Systems to Support Behavioral Interventions in Local Contexts: Scoping Review

**DOI:** 10.2196/77296

**Published:** 2026-02-25

**Authors:** Xi Yang, Martin Dijst, Joost G Daams, Marc Suhrcke, Haoran Yang, Ronald Cornet

**Affiliations:** 1 Department of Medical Informatics, Amsterdam Public Health Research Institute, Amsterdam UMC, University of Amsterdam, Meibergdreef 15, Amsterdam, 1105AZ, The Netherlands, 31 0205665205; 2 Department of Urban Development and Mobility, Luxembourg Institute of Socio-Economic Research, Esch-sur-Alzette, Luxembourg; 3 University of Luxembourg, Esch-sur-Alzette, Luxembourg; 4 Medical Library, Research Support, Amsterdam UMC, University of Amsterdam, Amsterdam, The Netherlands; 5 Department of Living Conditions, Luxembourg Institute of Socio-Economic Research, Esch-sur-Alzette, Luxembourg; 6 Centre for Health Economics, University of York, York, United Kingdom; 7 School of Geographic Sciences, East China Normal University, Shanghai, China; 8 Liwa Institute of Skin Health, East China Normal University, Shanghai, China

**Keywords:** health information system, behavioral intervention, environments, geo-referenced contexts, neighborhood geo-referenced contexts

## Abstract

**Background:**

Individual-level behavioral interventions are designed to improve health behaviors and manage noncommunicable diseases. Neighborhood geo-referenced contexts (NGRCs) significantly impact the success of these interventions. Integrating NGRC data into health information systems (HISs), including electronic medical records (EMRs), electronic health records (EHRs), and personal health records (PHRs), can enhance personalized NGRC-focused behavioral interventions and improve health outcomes. Despite the potential benefits, there is a notable gap in the literature about NGRC-focused behavioral interventions using HISs.

**Objective:**

This scoping review aims to review the current status and stakeholder insights of NGRC-focused behavioral interventions using HISs.

**Methods:**

Two reviewers examined publications indexed by MEDLINE (Ovid) and Scopus. Publications reporting on NGRC-focused behavioral interventions using HISs were included. We extracted data on study characteristics, population attributes, diseases, health setting characteristics, HISs, NGRCs, behavioral interventions, outcomes, and conclusions, with suggestions for future research.

**Results:**

The literature search identified 24 studies for inclusion. Of these, EHRs or EMRs were mostly used in 20 studies, with limited focus on PHRs. HISs were not used in the entire process of NGRC-focused behavioral interventions in 23 studies. Behavioral interventions in 21 studies focused exclusively on social needs interventions, with comparatively limited attention given to lifestyle change interventions. NGRCs mainly focused on built environments (n=24), with less attention to natural (n=1) and social (n=4) environments. The spatiotemporal characteristics of NGRCs were described in limited detail. “Destination accessibility” was the main focus within built environments. Other characteristics (“density,” “design,” “diversity,” and “distance”) were less studied. Stakeholders’ perspectives highlighted the value, convenience, patient autonomy, and privacy of integrating content (NGRCs) and/or technology (HISs) into behavioral interventions, despite the persistence of certain social concerns. We summarized 15 recommendations in terms of NGRC-focused behavioral interventions using HISs.

**Conclusions:**

PHRs have the potential to actively integrate more diverse, dynamic, and/or high-resolution NGRCs. Additionally, PHRs can participate in the entire process of NGRC-focused behavioral interventions, particularly in outpatient lifestyle change interventions. Health care providers can support these interventions to help achieve public health goals and advance health equity.

## Introduction

Behavioral interventions are strategies and techniques to influence health-related behaviors at the personal, community, and public policy levels [1]. They may encourage individuals to quit smoking or to exercise regularly. At the community level, behavioral interventions aim to change behaviors by modifying the environments that support them [1]. For example, enhancing existing sidewalks with improved lighting, repainting bike lane markings, and adding benches can create a more enjoyable experience that encourages walking and cycling. At the public policy level, the government or private groups can share health information to encourage behavioral change [1]. The absence of individual-level behavioral interventions poses a significant challenge in managing noncommunicable diseases (NCDs), which account for 70% of global deaths annually [2], as effective interventions for NCDs require long-term engagement and multiple follow-ups to detect relapses or late effects as soon as possible [3].

Neighborhood geo-referenced contexts (NGRCs) have been identified as barriers or facilitators to the acceptance of, participation in, and adherence to individual-level behavioral interventions [4]. NGRCs refer to the physical and social environments of a location or locations along one’s daily activities, and they influence people’s health-related behaviors [5-8]. NGRCs are a spatial manifestation of the social determinants of health (SDoH), which refer to the conditions in which people are born, grow, live, work, and age, as well as the broader systems and forces that shape these daily life conditions [9]. Beyond the social and natural environments in NGRCs, the built environment is characterized using the 5D framework, which includes *density*, *diversity*, *design*, *distance*, and *destination accessibility*. The failure to address NGRCs is evident from the focus on limited prevention programs or resources in health care settings aimed at addressing individual attitudes and behaviors, along with the limited resources for preventive care [10]. NGRC-focused behavioral interventions allow health care providers to tailor interventions to specific contexts, offering a more personalized approach. These interventions can be categorized into social needs interventions (eg, arranging transportation) and individual lifestyle change interventions (eg, walking in a nearby park). To achieve NGRC-focused behavioral interventions, studying the interaction between NGRCs and modifiable health-related behaviors is essential [11-13].

Health information system (HIS) data have been used to investigate the relationships between NGRCs and health behaviors among health providers, HIS vendors, payers, and researchers [14-24]. A HIS is a system for collecting and processing data, information, and knowledge in health care settings [25]. The category includes (1) provider-centric electronic medical record (EMR) systems used within a single health care setting, (2) electronic health record (EHR) systems spanning multiple settings, and (3) patient-centric personal health record (PHR) systems. Among PHRs, standalone PHRs are not integrated with EMRs or EHRs, whereas tethered and interconnected PHRs are linked to EMRs or EHRs [25-27]. These systems enable various stakeholders, such as health providers, patients, and caregivers, to access and process multimodal data, such as diagnoses, medication use, treatments, NGRCs, and behavioral information.

At the individual level, HISs can enhance NGRC-focused behavioral intervention via comprehensive NGRC data capture and effective intervention prescription, monitoring, and assessment [3]. HISs can help collect NGRC data during routine clinical care, supplemented by patient-reported information, or through linkage with publicly available external data sources like geographic information systems. At the community and policy levels, HISs can support cost-effective, large-volume, and time-efficient assessments of large-scale public health efforts, such as environmental interventions to support behavioral changes, compared with traditional surveys [28].

Although integrating NGRCs with HISs is not new [29], many reviews on the functionalities and features of EHRs, EMRs, and PHRs lack clarity regarding NGRCs [30-44]. Additionally, several systematic reviews have investigated the linkage between NGRC data and clinical data sources (eg, EHRs) for population health research, but they lack specifics regarding behavioral interventions [45,46]. Recent systematic reviews on SDoH screening, linkage, extraction, analysis, and interventions within HISs, especially EHRs, similarly show limited attention to NGRCs and/or intervention-based approaches [47-50]. Given the increasing interest and existing research gap in using HISs to prepare, prescribe, monitor, and assess NGRC-focused behavioral interventions, we conducted, to our knowledge, the first scoping review of the literature in this field to distinguish it from broader SDoH-focused reviews and those lacking an intervention-based approach [46,50]. This review excluded standalone PHRs, as they are independent of provider-centric EHRs or EMRs.

This scoping review aimed to answer the following questions:

1. To what extent and what types of HISs have been used in NGRC data capture, intervention prescriptions, monitoring, and assessments of NGRC-focused behavioral interventions, and across the 4 stages?
2. What types, spatiotemporal characteristics, and databases of NGRCs have been utilized in each type of behavioral intervention supported by HISs?
3. What are stakeholders’ insights regarding the use of HISs and/or NGRCs in behavioral interventions?
4. What are the gaps in research on the use of NGRCs and HISs in behavioral interventions?

## Methods

### Checklist

The analysis adhered to the PRISMA-ScR (Preferred Reporting Items for Systematic Reviews and Meta-Analyses extension for Scoping Reviews) checklist (Checklist 1) [51].

### Search for Relevant Research

An information specialist from the Amsterdam Medical Center library (JGD) was consulted to help define key concepts and establish the evaluation framework, which included selecting the appropriate databases, relevant keywords, and MeSH terms. MEDLINE (Ovid) and Scopus were searched from inception on April 29, 2024, to include studies up to the search date.

The search strategy could be conceptually summarized as follows: ([GRCs] AND (([neighborhoods] AND ([chronic care model] OR ([HISs] AND [health behaviors]))) OR ([community health facilities] AND [health behaviors]))), involving the following four elements related to our research questions: (1) HISs, (2) geo-referenced contexts (GRCs), (3) neighborhoods, and (4) health behaviors. Detailed information regarding the search terms in Scopus is shown in Textbox 1. The inclusion and exclusion criteria are presented in Textbox 2. For search terms in MEDLINE, refer to Table S2 in Multimedia Appendix 1.

Textbox 1. **Search terms in Scopus.**

[GRCs] AND (([neighborhoods] AND ([chronic care model] OR ([HISs] AND [health behaviors]))) OR ([community health facilities] AND [health behaviors]))

(((TITLE-ABS-KEY((("electronic health" or personal or digital or medical or patient or participant) W/1 record) OR (("electronic health" or personal or digital or medical or patient or participant or subject or adult) W/2 data) OR ehr or referral OR ((screen or screening) W/4 ("social determinant" or "social need" or sdoh))) OR ABS("clinical data" or "clinical information" or "clinical record")) AND TITLE-ABS-KEY(((socio or socioeconomic or social or psychosocial) W/0 (risk or stress or vulnerable or vulnerability or disadvantage or status)) or (((socio or socioeconomic or social or psychosocial) W/1 (need or environment)) or "health need") or ((socio or socioeconomic or social or psychosocial) W/2 (determinant or exposure or factor or problem)) or sdoh or hrsn or health equity or patient contextual or "life circumstance" or (socioeconomic W/0 (factor or deprivation or status)) or (socio W/1 (factor or deprivation or status)) or (community and (prevalence or risk)) or (community W/1 (type or context or factor or health*)) or (environment* PRE/0 (factor or design)) or ((community or environment*) W/2 exposure)) AND (TITLE-ABS-KEY(("residence characteristic" or "place of birth" or "birth place" or domicile or "health service area" or "catchment area" or neighbourhood or housing or census or urban or rural or county or counties or household) OR geocode or geocoding or "geo-code" or "geo-coding" OR (geograph W/2 (code or coding or data or divers or distribute or distribution or disparity or inequity or information or measure or measurement or record or residence)) OR ((patient W/0 context) or (contextual W/1 data)) OR ((demograhic W/2 (data or information or record)) or census) OR ((area or community) W/2 (factor or resource or research or impact or level or link or linked or linking or linkage)) OR (social W/2 information)) or ABS((demograhic W/3 factor) OR ((regional or region) W/9 patient) OR ((personal or individual) W/1 (sdoh or "social determinant")) OR "family centered" OR (social W/2 (risk or problem or factor)) OR geography or geographies)) OR (TITLE-ABS-KEY(((socio or socioeconomic or social or psychosocial) W/0 (risk or stress or vulnerable or vulnerability or disadvantage or status)) or (((socio or socioeconomic or social or psychosocial) W/1 (need or environment)) or "health need") or ((socio or socioeconomic or social or psychosocial) W/2 (determinant or exposure or factor or problem)) or sdoh or hrsn or health equity or patient contextual or "life circumstance" or (socioeconomic W/0 (factor or deprivation or status)) or (socio W/1 (factor or deprivation or status)) or (community and (prevalence or risk)) or (community W/1 (type or context or factor or health*)) or (environment* PRE/0 (factor or design)) or ((community or environment*) W/2 exposure)) AND TITLE-ABS-KEY((community W/1 (register or registry or data or record or program or provider)) or "community health center" or "community mental health center"))) AND (TITLE-ABS-KEY(((health or unhealth) PRE/1 behavior) or ((healthy or unhealthy) PRE/1 behavior) or smoking or tobacco or diet or food or nutrition or lifestyle or alcohol or "physical activit" or sedentary or (("social determinant" or SDOH) W/0 (screen or screening))))) OR (( TITLE-ABS-KEY ( "chronic care model" ) ) AND ( TITLE-ABS-KEY ( ( "residence characteristic" OR "place of birth" OR "birth place" OR domicile OR "health service area" OR "catchment area" OR neighbourhood OR housing OR census OR urban OR rural OR county OR counties OR household ) OR geocode OR geocoding OR "geo-code" OR "geo-coding" OR ( geograph W/2 ( code OR coding OR data OR divers OR distribute OR distribution OR disparity OR inequity OR information OR measure OR measurement OR record OR residence ) ) OR ( ( patient W/0 context ) OR ( contextual W/1 data ) ) OR ( ( demograhic W/2 ( data OR information OR record ) ) OR census ) OR ( ( area OR community ) W/2 ( factor OR resource OR research OR impact OR level OR link OR linked OR linking OR linkage ) ) OR ( social W/2 information ) ) OR ABS ( ( demograhic W/3 factor ) OR ( ( regional OR region ) W/9 patient ) OR ( ( personal OR individual ) W/1 ( sdoh OR "social determinant" ) ) OR "family centered" OR ( social W/2 ( risk OR problem OR factor ) ) OR geography OR geographies ) ) AND ( TITLE-ABS-KEY ( ( ( socio OR socioeconomic OR social OR psychosocial ) W/0 ( risk OR stress OR vulnerable OR vulnerability OR disadvantage OR status ) ) OR ( ( ( socio OR socioeconomic OR social OR psychosocial ) W/1 ( need OR environment ) ) OR "health need" ) OR ( ( socio OR socioeconomic OR social OR psychosocial ) W/2 ( determinant OR exposure OR factor OR problem ) ) OR sdoh OR hrsn OR health AND equity OR patient AND contextual OR "life circumstance" OR ( socioeconomic W/0 ( factor OR deprivation OR status ) ) OR ( socio W/1 ( factor OR deprivation OR status ) ) OR ( community AND ( prevalence OR risk ) ) OR ( community W/1 ( type OR context OR factor OR health* ) ) OR ( environment* PRE/0 ( factor OR design ) ) OR ( ( community OR environment* ) W/2 exposure ) ) ))

Textbox 2. **Inclusion and exclusion criteria.**

**Inclusion criteria**

- Study type: Original empirical research studies.
- Health information systems (HISs) and behavioral interventions: Used HISs to prepare, prescribe, monitor, and/or assess behavioral interventions.
- Neighborhood geo-referenced contexts (NGRCs): Used one or more NGRC attributes about the outside world to assist and/or adjust behavioral interventions.
- NGRCs and behavioral interventions: Studies in which NGRC and intervention data pertain to concurrent or longitudinal periods. Studies in which NGRC attributes and intervention attributes use either objective or subjective measurements.

**Exclusion criteria**

- Study type: Study protocols without substantive findings and studies without results (eg, experimental descriptions and commentaries).
- HISs: Studies for which it cannot be confirmed whether the records are part of an HIS due to an unspecified format (electronic or paper), or it is not explicitly stated whether these data are connected to HISs. Studies on standalone personal health records managed solely by patients and/or caregivers without the integration of data in medical settings (eg, hospitals). Studies using an HIS that was not focused on interventions but was used as a database to identify participants for empirical studies.
- NGRCs: Studies focusing only on broad geographic categories (eg, county, city, region, nation, and urban/rural), using sensors without specific attributes, NGRCs about health services, or economic factors (eg, transportation reimbursement). Studies for which it cannot be determined whether NGRCs were involved due to a lack of specific details on social determinants of health screening tools and interventions.

## Results

### Search Term Coverage and Rationale

GRCs include (1) built environments from the 5D dimensions (density, diversity, design, distance, and destination accessibility); (2) natural environments; and (3) social environments, which encompass neighborhood socioeconomic status and neighborhood social relationships. Neighborhoods include (1) static administrative areas, (2) various delineations surrounding each participant’s address, (3) perceived neighborhoods, and (4) geographic areas encompassing all locations visited by an individual in their daily activities. For the definitions and examples of NGRCs and neighborhoods, see Table S1 in Multimedia Appendix 1 [1,9,26,52-76]. We used 3 different search strategies because some concepts can be merged and replaced by related ones. For example, HISs and health behaviors are 2 critical elements of the chronic care model, which is a valuable framework for patient empowerment, supporting self-management and enhancing health outcomes [53-56]. Consequently, substituting HISs and health behaviors with search terms pertinent to the chronic care model while connecting them with GRCs and neighborhoods constituted the second part of the search strategy. Additionally, community health facility workers may use HISs to address neighborhood-related issues. Therefore, replacing HISs and neighborhoods with search terms relevant to community health facilities (eg, community health centers and community data) while linking these terms with GRCs and health behaviors formed the third part of our search strategy.

### Eligibility Criteria

All original peer-reviewed scientific studies in English indexed up to April 29, 2024, were included, while non–peer-reviewed studies and gray literature were excluded. Textbox 2 outlines the inclusion and exclusion criteria.

### Selection Process

First, we screened titles and abstracts and excluded studies according to the inclusion and exclusion criteria. When uncertainty arose, studies were retained for the full-text screening stage. Second, XY and MD independently conducted a full-text review and extracted the relevant NGRCs, HISs, and behavioral interventions from the potentially included papers. Any disagreements were first addressed by referring to a predefined inclusion/exclusion checklist. If consensus was not reached using the checklist, the reviewers discussed each case, and, if necessary, a third reviewer (RC) made the final decision. Although interrater reliability was not calculated, this structured approach ensured consistent and transparent decision-making.

### Data Extraction

We extracted data from the included studies using a 7-item table. These seven items included: (1) study attributes; (2) population attributes, diseases, and health setting characteristics; (3) HISs; (4) NGRCs; (5) behavioral interventions; (6) results; and (7) conclusions and future studies. Table S3 in Multimedia Appendix 1 provides further details.

**Figure 1.**
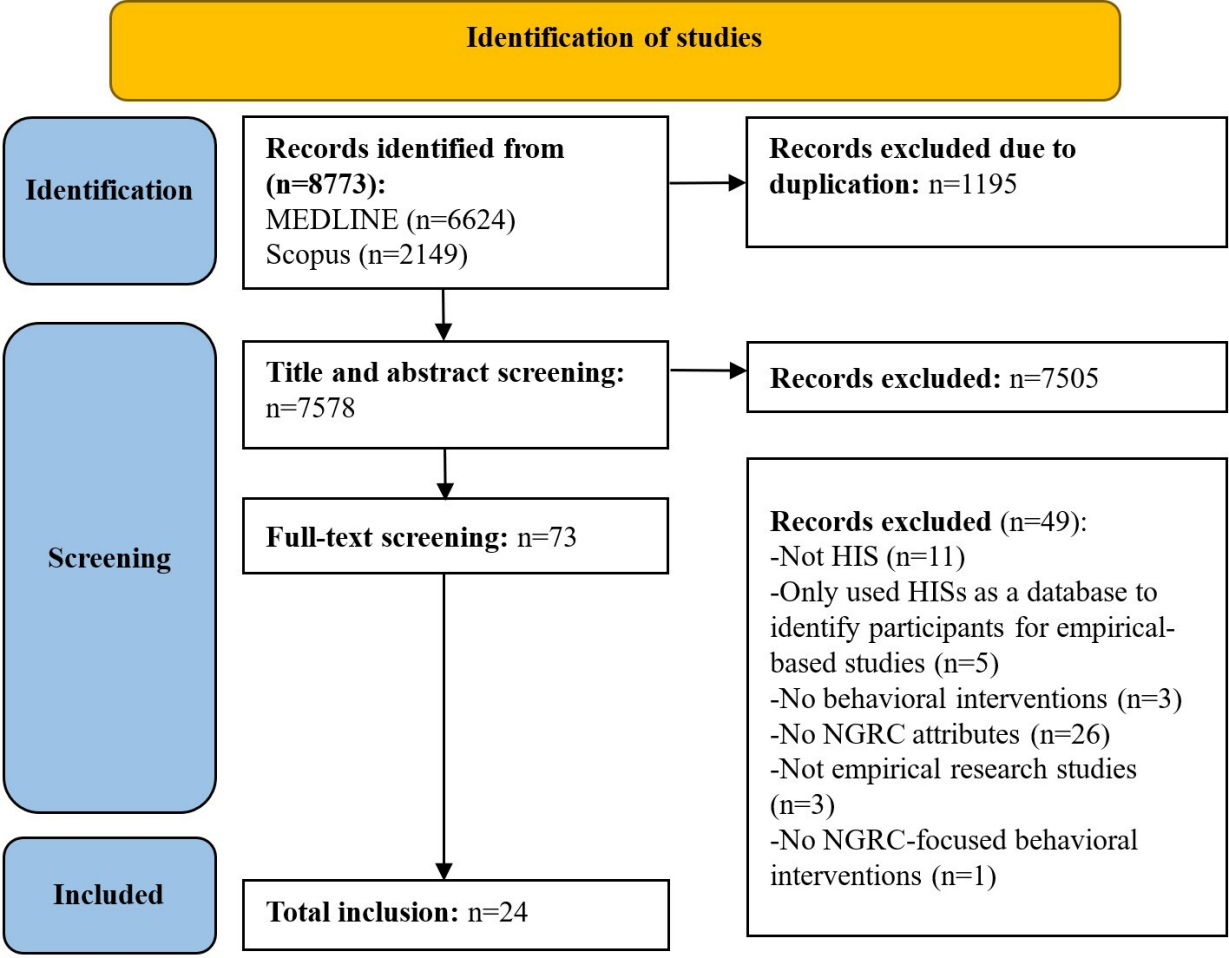
PRISMA (Preferred Reporting Items for Systematic reviews and Meta-Analyses) flowchart. HIS: health information system; NGRC: neighborhood geo-referenced context.

Figure 1 presents the PRISMA flowchart, which includes the identification, screening, and selection process. A total of 24 peer-reviewed studies were finally included.

### Study Characteristics

We reviewed the descriptive characteristics of all 24 included papers. Basic characteristics of the studies are presented in Table S4 in Multimedia Appendix 1, including information on (1) the type, number, gender, age distributions, and language of participants, and (2) the study type and year of publication. The characteristics with appropriate properties for summarization include (1) the type, NCD, and health and computer literacy of participants, and (2) health settings. All studies were conducted in the United States. Details are provided in Table S5 in Multimedia Appendix 1.

We divided the studies by participant type as follows: 2 studies [29,77] focused solely on providers, 4 studies concentrated on caregivers [78-81], 13 studies examined patients only [10,82-93], and 5 studies involved both health providers and patients [93-97]. Among patients, most studies focused on adults.

### Summarization of the Results

#### Overview

In this section, we aim to summarize the findings from the 24 included articles to address the first 3 research questions. Each of these themes is explored in detail in the subsections that follow.

#### Question 1: Question Regarding HISs

We summarized whether HISs were used and the types of HISs used. Table 1 presents HIS usage across all stages, while Table S5 in Multimedia Appendix 1 lists the types of HISs. Table S4 in Multimedia Appendix 1 provides the specific names of the HISs.

Table 2 details the stage 1 NGRC data capture, including the settings (eg, health care settings, such as hospitals, and non–health care settings, such as homes), those collecting data (eg, patients, providers, and systems), and those documenting it (systems or providers).

**Table 1.** The role of HISs^a^ in the entire process of NGRC^b^-focused behavioral interventions, including situations and actors.

Group and study	NGRC data capture (n=18)	Prescription (n=13)				Monitoring (n=2)	Assessment (n=11)
		Digital prescriptions (n=6)		Prescriptions by HPs^c^ and documented in HISs (n=7)			
		Tools	Interventions	Types of HPs	Interventions		
Social needs interventions							
Buitron de la Vega et al [82]	HISs^d^	EHR^d^^,^^e^	Referral guides^d^	—^f^	—	—	—
Cordova-Ramos et al [78]	HISs^d^	—	—	Nurses^d^	—	—	—
Fortin et al [79]	HISs^d^	—	—	Social workers^d^	EHR-based resource maps^d^	—	—
Lynch et al [94]	HISs^d^	—	—	—	—	Manual documentation^d^	—
Palakshappa et al [95]	HISs^d^	A tablet-based patient portal^d^	CRLs^g^ and TIAtHPs^d^^,^^h^	—	—	—	—
Percac-Lima et al [85]	HISs^d^	—	—	—	—	—	The percentage of CRC^i^ screening^d^
Russell et al [84]	HISs^d^	—	HISs^d^	Nurses^d^	—	—	—
Wallace et al [86]	—	—	—	—	—	—	HCUD^d^^,^^j^
Wright et al [96]	HISs^d^	—	HISs^d^	Social workers^d^	—	—	—
Wu et al [97]	HISs^d^	—	—	—	—	—	HCUD and medication adherence^d^
Roth et al [88]	—	—	—	—	—	—	HCUD, HbA_1c_ levels, and blood pressure^d^
Tanumihardjo et al [87]	HISs^d^	—	—	—	—	—	HbA_1c_, LDL^k^, and blood pressure^d^
Holt et al [29]	HISs^d^	Web-based PatientWisdom patient portal^d^	Resource linkages and TIAtHPs^d^	—	—	—	—
Bechtel et al [89]	—	—	—	—	—	—	HCUD^d^
Taber et al [90]	HISs^d^	—	—	—	—	—	—
Stark et al [80]	HISs^d^	Healthy Planet app^d^	CRLs^d^	—	—	—	—
Gupta et al [91]	HISs^d^	NowPow embedded in EMR^d^^,^^l^	Resource linkages^d^	—	—	Automatic documentation^d^	HCUD^d^
Britto et al [92]	HISs^d^	—	—	Researchers^d^	HISs^d^	—	The percentage of patients with well-controlled asthma^d^
Glasser et al [81]		EHR-integrated CommunityRx^d^	Resource linkages^d^	—	HISs^d^	—	—
Loo et al [93]	HISs^d^	—	—	EDCS^d^^,^^m^	HISs^d^	—	—
Percac-Lima et al [98]	HISs^d^	—	—	Navigators^d^	HISs^d^	—	HCUD^d^
Lifestyle change interventions							
Jilcott et al [10]	—	—	—	—	—	—	HCUD, blood lipids, glucose, pressure, BMI, and body composition^d^
Johnston et al [83]	—	—	—	—	—	—	HCUD^d^
The entire care process							
Kane et al [77]	HISs^d^	—	—	—	—	—	—

a HIS: health information system.

b NGRC: neighborhood geo-referenced context.

c HP: health provider.

d HISs have functioned in this category.

e EHR: electronic health record.

f Not applicable.

g CRL: community resource list.

h TIAtHP: timely intervention alert to health provider.

i CRC: colorectal cancer.

j HCUD: health care utilization data.

k LDL: low-density lipoprotein.

l EMR: emergency medical record.

m EDCS: emergency department clinical staff.

**Table 2.** The role of HISs^a^ in NGRC^b^ data capture.

Group and study	NGRC preparation (n=18)								
	H/NH^c^	NGRC data collection (n=18)						NGRC data documentation (n=18)	
		Patient-administered electronic collection (n=5)		Patient-administered paper-based collection (HPs^d^ to assist if needed) (n=2)	HP-administered collection (n=10)		System-administered collection (n=1)	System (n=5)	HPs (n=13)
		Tools	Types of HPs		V/E^e^	Types of HPs			
Social needs interventions									
Buitron de la Vega et al [82]	H	—^f^	—	Medical assistants^g^	—	—	—	—	Medical assistants^g^
Cordova-Ramos et al [78]	H	—	—	—	V^g^	Nurses^g^	—	—	Nurses^g^
Fortin et al [79]	H	REDCap^g^	—	—	—	—	—	—	CTAs^g^^,^^h^
Lynch et al [94]	H	—	—	—	E^g^	Medical assistants^g^	—	—	Medical assistants^g^
Palakshappa et al [95]	H	Tablet-based patient portal^g^	—	—	—	—	—	HISs^g^	—
Percac-Lima et al [85]	H	—	—	—	V^g^	Navigators^g^	—	HISs^g^	Navigators^g^
Russell et al [84]	H	eScreening platform^g^	CTAs^g^	—	—	—	—	—	—
Wright et al [96]	H	—	—	Nurses^g^	—	—	—	—	Nurses^g^
Wu et al [97]	H	—	—	—	V^g^	Pharmacists^g^	—	—	Pharmacists^g^
Tanumihardjo et al [87]	H	—	—	—	V^g^	CHWs^g^^,^^i^	—	—	CHWs^g^
Holt et al [29]	NH	Web-based PatientWisdom patient portal^g^	—	—	—	—	—	HISs^g^	—
Taber et al [90]	H	—	—	—	V^g^	Pharmacists, nurses, social workers, and CHWs^g^	—	—	Pharmacists, nurses, social workers, and CHWs^g^
Stark et al [80]	NH	Healthy Planet app^g^	—	—	—	—	—	HISs^g^	—
Gupta et al [91]	H	—	—	—	E^g^	Without specificity^g^	—	—	Without specificity^g^
Britto et al [92]	H	—	—	—	V^g^	Researchers^g^	—	—	Researchers^g^
Loo et al [93]	H	—	—	—	V^g^	Nurses and clinicians^g^	—	—	Nurses^g^
Percac-Lima et al [85]	H	—	—	—	V^g^	Navigators^g^	—	—	Navigators^g^
Entire care process									
Kane et al [77]	H	—	—	—	—	—	Publicly available datasets^g^	HISs^g^	—

a HIS: health information system.

b NGRC: neighborhood geo-referenced context.

c H/NH: physical health settings (H), and non–health care settings (NH).

d HP: health provider.

e V/E: verbal surveys embedded in electronic health records (EHRs) or electronic medical records (EMRs) (E), or surveys not embedded in EHRs or EMRs (V).

f Not applicable.

g HISs have functioned in this category.

h CTA: care team assistant.

i CHW: community health worker.

#### Question 2: Question Regarding NGRCs

We summarized the reviewed studies in terms of behavioral intervention types as follows: (1) social needs interventions, (2) lifestyle change interventions, and (3) whole care without specifying the behavioral intervention type. This categorization agrees with the information in Table S1 in Multimedia Appendix 1. Table 3 summarizes the types and spatiotemporal characteristics of NGRC attributes.

**Table 3.** The types and spatiotemporal characteristics of NGRCs^a^ related to social needs interventions, lifestyle interventions, and the entire care process.

Group and NGRC	B/N/S^b^	Neighborhood type (address)	Data source^c^	SDoH^d^ screening	Ob/Su^e^	I/O^f^	Li/So/E^g^	Reference
Social needs interventions (n=21)								
Transportation barriers (inpatient)	B	PNs^h^	Sc	Yes	Su	I	So	[78,79,82,84-92,94-96,98]
Transportation barriers (outpatient)	B	PNs	Sc	Yes	Su	O	So	[29,80,81,93,97]
Neighborhood safety (individuals' fears and feelings of insecurity in their environment)	S	PNs	Sc	Yes	Su	I	So	[90]
Lifestyle interventions (n=2)								
Design: sidewalk/bike lane shortage	B	PNs (RDs^i^)	—^j^	Yes	Su	I	So	[10]
Density: intensive fast foods, insufficient healthy food options (eg, food stores, restaurants, farmer’s markets, and fruit stands), and healthy breakfast access	B	PNs (RDs)	—	Yes	Su	I	So	[10]
Density: recreational facility scarcity for walking (eg, parks, gyms, and tracks), affordable exercise facilities, and suitable physical activity programs	B	PNs (RDs)	—	Yes	Su	I	So	[10]
Traffic	B	PNs (RDs)	—	Yes	Su	I	So	[10]
Design: lack of streetlights	B	PNs (RDs)	—	Yes	Su	I	So	[10]
Distance to a high-risk location, such as a bar or supplier the individual used to frequent	B	Daily routes	L	No	Ob	O	Li	[83]
Living in an environment where alcohol use is prevalent or normalized	S	PNs (RDs)	Sc	No	Su	O	Li	[83]
Community crime	S	PNs (RDs)	Sc	No	Su	I	So	[10]
Verbal abuse from people on the street and speeding drivers	S	PNs (RDs)	Sc	No	Su	I	So	[10]
Air pollution from cars or factories	N	PNs (RDs)	Sc	No	Su	I	So	[10]
The entire care process (n=1)								
Food desert status (yes/no) and low food access census tract (yes/no)	B	Census tracts (RDs)	P	No	Ob	I	E	[77]
Location type (urban/rural)	B	Census tracts (RDs)	P	No	Ob	I	E	[77]
Total population, low-income population, children aged 0-17 years, seniors aged 65 years or older, and housing units lacking vehicle access among populations with limited access, defined by how far the closest supermarket is (0.5, 1, 10, and 20 miles away)	S	Census tracts (RDs)	P	No	Ob	I	E	[77]
Community-level socioeconomic and demographic data, including age, race, ethnicity, nationality distribution, median age, median family and household income, education level distribution, median home value and gross rent, renter-occupied household distribution, total population, total housing units, and low-income census tract (yes/no)	S	Census tracts (RDs)	P	No	Ob	I	E	[77]

a NGRC: neighborhood geo-referenced context.

b B/N/S: built environment (B), natural environment (N), or social environment (S).

c Source: NGRC screening (Sc), location-aware devices (L), or public datasets (P).

d SDoH: social determinants of health.

e Ob/Su: objective (Ob) or subjective (Su).

f I/O: inpatient (I) or outpatient (O).

g Li/So/E: lifestyle change interventions (Li), social needs interventions (So), or entire care process (E).

h PN: perceived neighborhood.

i RD: residential address.

j Not available.

We specified substructures for the categorization of social needs interventions, lifestyle change interventions, and whole care. Social needs interventions used actively collected, subjective, and static measures of perceived neighborhood social and built environments. Lifestyle change interventions included (1) actively collected, subjective, and static measures of perceived neighborhood social, natural, and built environments at residential addresses, and (2) passively collected, objective, real-time, and location-based distances to high-risk areas (eg, bars). Whole care included passively collected, objective, and static social and built environment indicators derived from the census tract of residential addresses.

In social needs interventions, actively collected, subjective, and static measures of perceived transportation barriers and neighborhood safety were the only 2 assessed NGRCs (21/24, 88%). These were assessed using electronic or paper-based surveys [29,78-82,84-98]. The focus on transportation barriers (21/24, 88%) may reflect their relative ease of mitigation through interventions (eg, door-to-door transportation to medical appointments [8,92]). These interventions were shaped by specific health service settings and available community resources. Similarly, transportation barriers were assessed using a variety of question formats, time frames, destination types, and SDoH screening tools. Despite these efforts, detailed information, such as transport mode, distance, travel time, and the locations of perceived neighborhoods, was generally rarely collected. Additional details are presented in Table 4.

**Table 4.** Additional details regarding transportation barriers.

Questions about NGRCs^a^		Timespan	Survey frequency	SDoH^b^ screening tools	Reference
Transportation barriers to attending medical appointments (eg, CRC^c^ screening) or obtaining medications (n=10)					
Do you have trouble getting transportation to medical appointments?		—^d^	—	Tool for Health and Resilience In Vulnerable Environments Social Determinants of Health (THRIVE)	[82,93]
Do you have trouble with transportation to or from NICUs^e^? For example, paying for parking or getting a ride to the hospital.		—	—	Tool for Health and Resilience In Vulnerable Environments Social Determinants of Health (THRIVE)	[78]
Query regarding transportation barriers to CRC screening		—	—	—	[85]
Query regarding transportation barriers to CT^f^ screening tests (eg, lung cancer and chest)		—	—	—	[98]
Query regarding transportation to appointments		—	—	—	[92]
Have you missed any medical appointments because of difficulties with transportation?		Past 6 and 12 months	Annually	Health Risk Assessment (HRA)	[87]
Have you ever not seen a doctor because you did not have a way to get to the clinic or hospital?		Past 12 months	—	HealthLeads	[86]
Has a lack of transportation kept you from medical appointments or from getting medications? (query 1)		Past 12 months	—	Intensifying Community Referrals for Health (SINCERE)	[80]
Has a lack of transportation kept you from medical appointments or from getting medications? (query 2)		Past 3 months	—	Monteliore's 10-question SDoH survey	[91]
Transportation barriers to attending medical appointments, meetings, or work, or getting things needed for daily living (n=9)					
Has a lack of transportation kept you from medical appointments, meetings, or work, or from getting things needed for daily living? (query 1)		Past 12 months	—	Health-Related Social Needs (HRSN)	[79,95]
Has a lack of transportation kept you from medical appointments, meetings, or work, or from getting things needed for daily living? (query 2)		Past 12 months	—	Protocol for Responding to and Assessing Patients’ Assets, Risks, and Experiences (PRAPARE)	[87]
Has a lack of transportation kept you from medical appointments, meetings, or work, or from getting things needed for daily living? (query 3)		—	—	Protocol for Responding to and Assessing Patients’ Assets, Risks, and Experiences (PRAPARE)	[88,94]
Has a lack of transportation kept you from medical appointments, meetings, or work, or from getting things needed for daily living? (query 4)		—	—	Assessing Circumstances and Offering Resources for Needs	[84]
Has a lack of transportation kept you from work, attending medical appointments including dialysis treatments, or getting things you need for your daily living?		—	Monthly [96]; — [89]	The Core 5 Determinants of Health Screening Tool	[89,96]
Query regarding challenges in finding affordable, reliable, or handicap-accessible transportation to obtain resources such as medical care, food, or medications		—	—	—	[90]
Transportation barriers without specifying which destinations were affected (n=3)					
Query regarding transport		—	—	—	[29,81]
By the way, we have this new resource team that can help with things not related to health care, like food, housing, and transportation. Is this something you want to talk about?		—	Weekly	—	[97]

a NGRC: neighborhood geo-referenced context.

b SDoH: social determinants of health.

c CRC: colorectal cancer.

d Information not available.

e NICU: neonatal intensive care unit.

f CT: computed tomography.

Lifestyle change interventions assessed actively collected, subjective, and static measures of social, natural, and built environments at participants’ perceived residential neighborhoods. These were assessed with “neighborhood assessment” during follow-up visits [10]. These assessments showed the multifaceted interplay between NGRCs and lifestyle behaviors. The environmental barriers to healthy eating and physical activity included air quality, food environment density, physical activity facility density, streetlight design, and neighborhood safety (Table 3). The absence of subsequent similar studies highlights the practical challenges of consistently partnering with community groups and agencies to address environmental barriers to lifestyle change.

**Figure 2.**
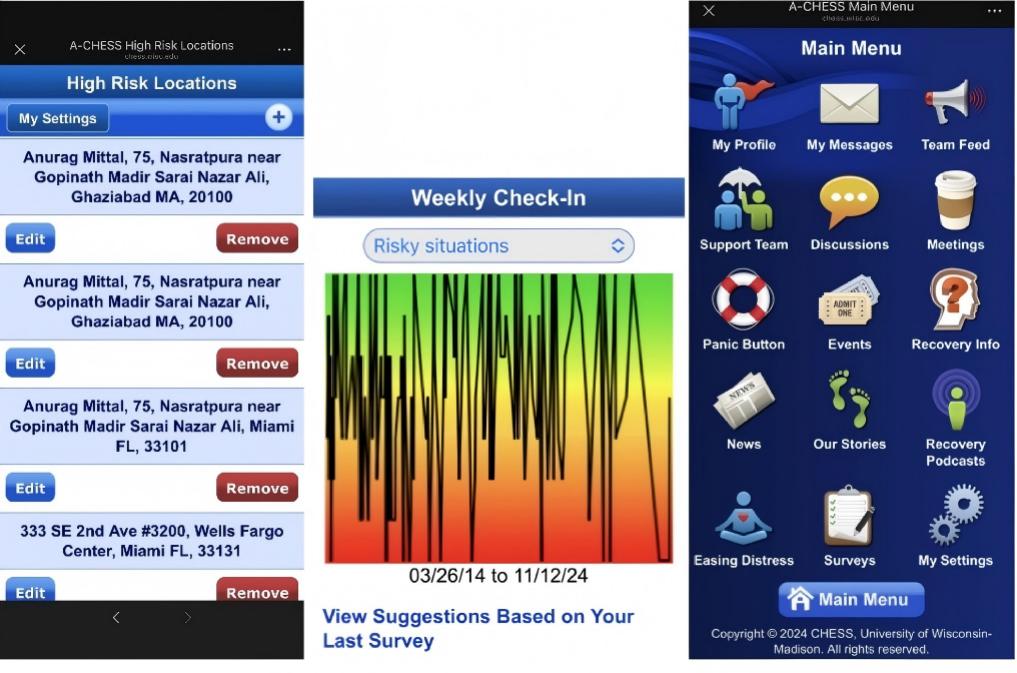
The A-CHESS (Addiction Comprehensive Health Enhancement Support System) app [83] supports recovery from alcohol use disorder by providing 24/7 smartphone access to services such as cognitive-behavioral therapy, addiction-related resources, and alcohol-free events. The left panel displays a list of identified high-risk locations, the middle panel summarizes risky situations based on weekly surveys, and the right panel shows the app’s homepage. Using GPS, A-CHESS monitors when users approach predesignated high-risk locations, such as bars, and sends an alert asking whether they intend to be there. No alert is issued if the user is not near a high-risk location. The screenshots were captured from the app link [99].

In addition, passively collected, objective, real-time, and location-based distances to high-risk areas (eg, bars) were assessed using GPS data from the A-CHESS (Addiction Comprehensive Health Enhancement Support System) app [83] (Figure 2). Unlike approaches that depend on community-level resources of residential addresses, A-CHESS optimizes users’ daily routes. This suggests that leveraging existing environments along these routes—not just around residential areas—can be practical without requiring extensive organizational collaboration. A-CHESS also supports weekly surveys to actively assess participants’ perceived alcohol use in their residential neighborhoods [83]. While transportation barriers are relatively stable, neighborhood social cultures, such as drinking behaviors, fluctuate rapidly, requiring active and higher-resolution data collection to capture their health effects.

In whole care, passively collected, objective, and static social and built environment indicators based on the census tract level of the residential address were displayed in the Envirome Web Service [77]. Integrated with the Cerner Millennium EHR, this service displays socioeconomic data, food desert status, and contextual information at the census tract level using public datasets (eg, the US Department of Agriculture and the 2010 Decennial Census). Compared with A-CHESS, the Envirome Web Service offers broader, population-level coverage of social and built environment factors through passive data collection but lacks dynamic, route-specific, or nonresidential contextual information.

#### Question 3: Question Regarding Stakeholders’ Insights

We summarized stakeholders’ insights on the use of NGRCs and/or HISs in social needs interventions and whole care from 10 included studies. The details are shown in Table S6 in Multimedia Appendix 1.

Seven studies mentioned attitudes toward NGRC integration. Six out of the seven studies on social needs interventions found that patients, caregivers, and providers valued addressing transportation barriers for comfort and convenience [84,93,96,97]. However, challenges remain in addressing patients’ concerns about potential discrimination, such as reduced courtesy or respect, as well as providers’ discomfort with stigmatizing questions [81,86,93,96,97]. At the core, the challenge lies in balancing actionable social needs interventions and sensitive care. The last study [77] examined providers’ views on NGRC data needs, clinical usefulness, and data visualizations in whole care. Providers emphasized the need for clear definitions and simple visualizations to make complex data more actionable in practice [77].

Nine studies examined HIS integrations from 3 aspects: value and convenience, patient autonomy, and privacy. The efficiency and convenience of electronic NGRC capture tools were recognized by providers, patients, and caregivers, particularly when patients completed them before visits [29,77,86,95-97]. However, providers also expressed concerns about increased workload, time constraints, unfitness to the workflow, and the possibility of raising unmet patient expectations [29,86,94-96]. These concerns underscore the need to integrate NGRC-focused behavioral interventions seamlessly into clinical workflows to avoid a higher administrative burden. Patient autonomy and engagement were enhanced [77,86,95-97]. Improved autonomy can support person-centered and team-based care. However, the absence of data on patients’ digital literacy could lead to misinterpretation of patients’ attitudes (eg, acceptability, adoption, and usage of HISs), especially for those with low literacy levels, as higher digital literacy can alleviate social concerns [29,84]. Privacy was improved by reducing patients’ feelings of judgment and promoting greater honesty in disclosure [84,86,95]. Improved privacy suggests that well-designed digital systems can mitigate stigma associated with sensitive NGRCs.

## Discussion

### Principal Findings and Recommendations

In this scoping review, we reviewed the current state of NGRC-focused behavioral interventions using HISs in order to propose recommendations for future studies. We included 24 studies, and nearly half of the studies (11/24, 46%) were conducted in 2023 or later (Table S4 in Multimedia Appendix 1). Due to unstructured reporting in many of the included studies, reliably extracting data was difficult. We organized our findings and recommendations according to the 4 research questions.

### Question 1: Question Regarding HISs

The predominant reliance on provider-centric EHRs/EMRs and the limited and inconsistently described use of HISs across the entire NGRC-focused behavioral intervention cycle significantly constrain the integration of NGRCs into behavioral interventions. Apart from the unspecified type of HIS in 1 study [10], most included studies relied on provider-centric EHRs/EMRs (20/24, 83%), with limited use of more patient-empowered PHRs. Only 3 studies used tethered PHRs, and none utilized interconnected PHRs (Table S5 in Multimedia Appendix 1). The reliance on provider-centric EHRs/EMRs may lead to difficulties in monitoring and assessment. Therefore, most studies (23/24, 96%) did not apply HISs in the entire process (NGRC data capture, intervention prescription, monitoring, and assessment). HISs were mainly used for (1) NGRC data capture (18/24, 75%), with limited patient-led (5/24, 21%) or system-led (1/24, 4%) cases; (2) intervention prescriptions (13/24, 54%); and (3) assessments (11/24, 46%) focusing on health care utilization, with minimal use in monitoring (2/24, 8%). Limited monitoring and a narrow focus on health care utilization data for assessments leave the sustainability of behavior change largely unaddressed. Given its critical role in improving long-term health outcomes, future research should aim to establish integrated frameworks that embed tethered or interconnected PHRs consistently across the 4 phases. Furthermore, the lack of details on HIS functionalities limits the ability to synthesize evidence and derive practical design recommendations for future interventions. Future studies should provide sufficient details to ensure reproducibility.

Although HISs have been used frequently in NGRC data capture, the dominance of active collection (eg, patient-administered or provider-administered surveys; 22/24, 92%) and the underuse of passive collection (eg, linkage with public datasets; 1/24, 4%) and GPS data (1/24, 4%) have left existing data underutilized, and active collection may cause user information to be unclear. On one hand, the majority of studies (17/24, 71%) relied on manual NGRC collection and documentation into EHRs/EMRs by stakeholders (Table S6 in Multimedia Appendix 1). Active data collection likely contributes to negative provider attitudes due to increased workload and limited consultation time. This potentially hinders the integration of NGRCs into routine care. Self-completed assessments using tethered or interconnected PHRs may address providers’ concerns, and both patients and providers hold positive attitudes toward them (Table S6 in Multimedia Appendix 1). However, tethered or interconnected PHRs were underutilized in the included studies. The gap between positive stakeholder attitudes and practice suggests missed opportunities to reduce provider burden and engage patients more actively in their care using PHRs. On the other hand, although the A-CHESS app achieved passive data collection, it relied solely on HISs as assessment tools and had limited provider access to behavioral data, which constrained the understanding of how HISs and providers influence behavior change and its sustainability. Similarly, the Envirome Web Service [77] passively collected NGRC data via public dataset linkages. However, the lack of detailed descriptions of the use of HISs in subsequent steps (eg, monitoring and assessment) further limited insights into their role in supporting sustained behavior change.

The complexity of provider types and roles in the entire process of NGRC-focused behavioral interventions (Table 1) further complicates implementation in clinical settings. Future studies should clearly define the responsibilities of every provider to enhance coordination across care teams, thereby improving intervention fidelity and consistency.

**Figure 3.**
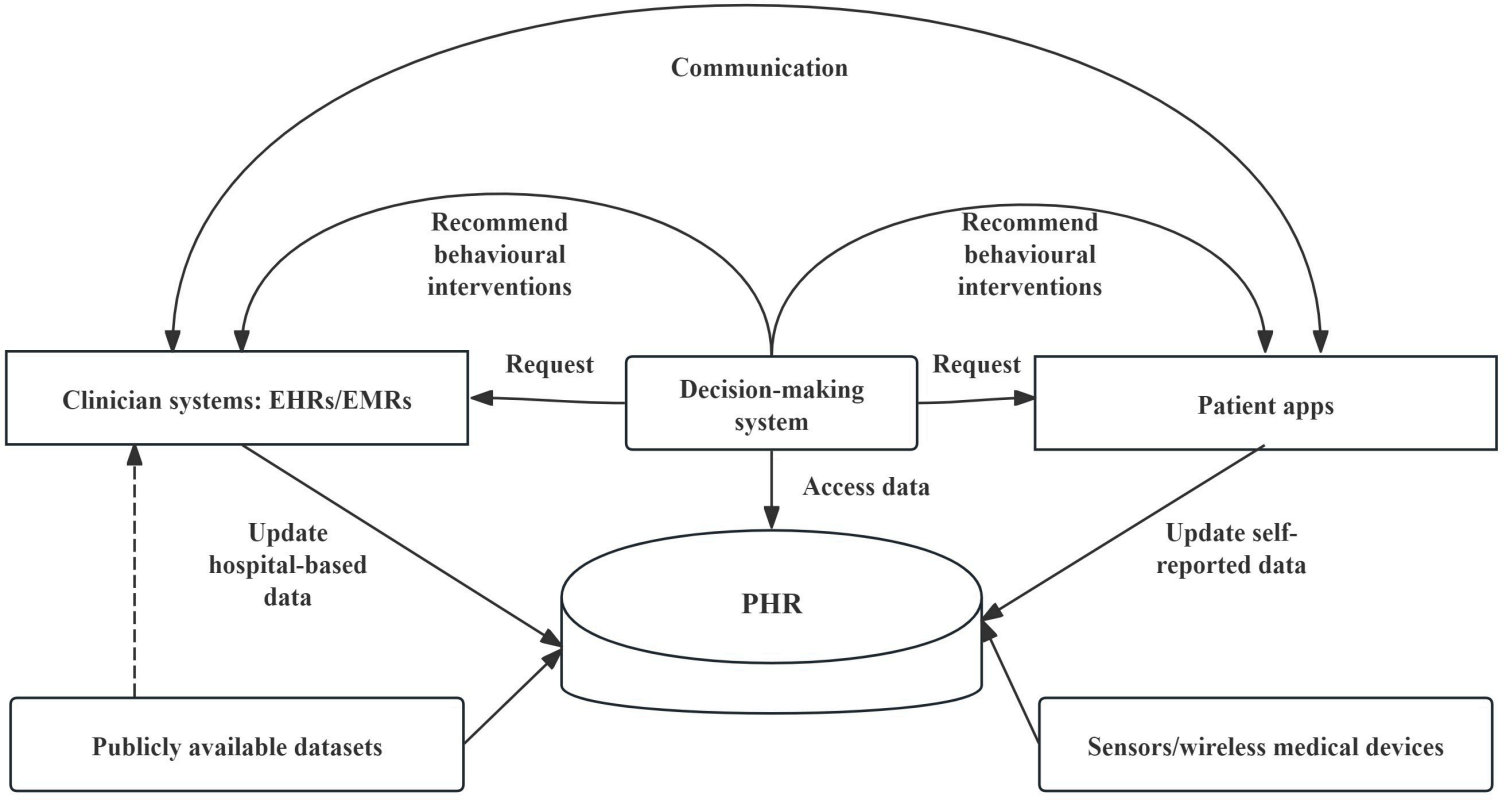
Tethered and interconnected advantages of PHRs in NGRC-focused behavioral interventions. The image has been modified [3]. EHR: electronic health record; EMR: electronic medical record; NGRC: neighborhood geo-referenced context; PHR: personal health record.

In summary, implementing tethered or interconnected PHRs throughout the intervention process, particularly in outpatient settings, is recommended (Figure 3). Connection with EHRs/EMRs can also achieve provider support. By ensuring privacy, security, and controlled access, PHRs can enhance user acceptance and usage [100]. Secure data exchange among patient apps, public datasets, sensors, and EMRs/EHRs can enable tailored interventions, timely issue detection, improved communication, greater engagement and adherence, better care coordination, higher satisfaction, and reduced treatment burden [86,101]. We have summarized 3 recommendations in Textbox 3.

Textbox 3. **Recommendations.**

**Recommendations regarding the types and roles of health information systems (HISs) in the entire process of neighborhood geo-referenced context (NGRC)–focused behavioral interventions**

1. Consider tethered or interconnected personal health records (PHRs), especially in outpatient settings, in the entire process of NGRC-focused behavioral interventions.
  - Use tethered or interconnected PHRs to actively or passively collect multiple NGRCs and generate a more comprehensive view of patients’ NGRCs [84]. PHRs can passively obtain location-related NGRCs through connections with electronic health records (EHRs)/electronic medical records (EMRs), public datasets (eg, government data and cohorts), sensors, and wireless medical devices. The locations can include not only the residential address but also regularly visited places or GPS-derived data. On the other hand, PHRs can also embed patient- or caregiver-completed surveys to actively collect perceived NGRCs. Determining the optimal timing and frequency of data capture is essential to balance user burden and data quality [82,84,102,103].
  - Clinical decision support systems (CDSSs) can use NGRC data from PHRs to personalize behavioral interventions, delivering recommendations to patients via apps and to providers via EHRs/EMRs. For instance, real-time CDSS alerts sent at optimal intervals can support providers in adjusting NGRC-focused interventions.
  - Consider integrating PHRs with other portable devices (eg, patient apps, sensors, and wireless medical devices) to allow real-time monitoring through surveys, GPS, and sensors.
  - Integrating PHRs with EHRs/EMRs and other portable devices can provide sufficient long-term follow-up data. CDSSs can then use these data to assess and adapt NGRC-focused behavioral interventions [46,85,104], allowing the evaluation of the sustainability of outcomes and the identification of interventions that produce lasting effects.
2. Incorporate the support of health care providers throughout NGRC-focused behavioral interventions. This support can be provided through (1) providers’ guidance on CDSS recommendations and (2) direct patient-provider communication.
  - Explore the roles of various health care providers in the entire process of NGRC-focused behavioral interventions.
3. Balance active and passive NGRC data collection to achieve higher acceptability, feasibility, and effectiveness of NGRC-focused behavioral interventions [24,84,86].

**Recommendations regarding the types, spatiotemporal characteristics, and datasets of NGRCs in each type of behavioral intervention**

1. Accommodate NGRC-focused social needs interventions with lifestyle change interventions.
2. Include a broader range of NGRCs with detailed spatiotemporal information for different behavioral interventions.
  - Expand the focus beyond influential built environments to also include key natural and social environments for specific behaviors.
  - Incorporate a comprehensive 5D (density, diversity, design, distance, and destination accessibility) framework of built environments.
  - Optimize NGRC spatiotemporal resolution using an evolutionary approach. Apply high-resolution or real-time data when available for precise interventions; otherwise, use static or coarse data (eg, administrative datasets) to capture neighborhood influences. Combining dynamic and static NGRCs can mitigate challenges such as the modifiable areal unit problem, the uncertain geographic context problem, and neighborhood effect averaging.
  - Prioritize key subjective and objective NGRCs for specific behaviors (eg, cycling and walking) and standardize their definitions, spatiotemporal attributes, and measurements through multidisciplinary collaboration. Such efforts should leverage and expand existing terminologies (eg, “community details” in Systematized Nomenclature of Medicine—Clinical Terms [SNOMED CT] and Logical Observation Identifiers Names and Codes [LOINC]) and ontologies (Social Determinants of Health Ontology [SOHO] [105] and social determinants of health ontology [SDoHO] [106]) [24].
  - Assess the availability of influential objective NGRCs in existing datasets and explore opportunities to collect missing objective and subjective data through passive methods (eg, sensors). When certain NGRCs cannot be captured passively, develop self-administered questionnaires to obtain the remaining information. Finally, evaluate the quality, usability, and improvement potential of patient-completed data.
3. Address both technical and social considerations (eg, ethical issues) in NGRC data collection [92].

**Recommendations regarding stakeholders’ insights on the use of HISs and/or NGRCs in behavioral interventions**

1. Assess stakeholder acceptance, attitude, and preferred interactive features for various NGRC-focused behavioral interventions using HISs, particularly those targeting lifestyle changes. For example, evaluate patients’ willingness and desired features in PHR-based self-management tools, and design EHR/EMR features that fit providers’ workflows to mitigate concerns like time constraints and workload.
2. Develop and iteratively test efficient, user-friendly data visualization tools for stakeholders [33,107-111].
3. Explore stakeholders’ perspectives on integrating diverse and/or real-time NGRCs into behavioral interventions, extending beyond transportation barriers to broader applications. Future research should identify which NGRCs stakeholders consider most actionable and relevant for specific interventions, and determine effective, nonstigmatizing data collection methods. For example, assess patient willingness to share GPS data when they can control access to their information.
4. Consider stakeholders’ computer and health literacy when evaluating their attitudes toward NGRC-focused behavioral interventions using HISs.
5. Iteratively build support systems, responding to negative feedback by aligning patient expectations and addressing stakeholders’ social concerns.

**Recommendations regarding research designs**

1. Adopt standardized and detailed descriptions of NGRCs [24], HIS functionalities, intervention designs, and relevant disease contexts—ideally guided by common metadata standards or consensus-based frameworks—to enhance transparency, comparability, and collaboration across studies.
2. Evaluate the feasibility, acceptability, adoption, sustainability, effectiveness, cost-effectiveness, ethics issues, and mechanisms of change of NGRC-focused behavioral interventions using HISs, with detailed descriptions of research design for reproduction, testing, and comparison. For example, in research design, the only difference between the intervention and control groups should be whether the intervention is NGRC-focused, in order to isolate the effect of NGRCs.
3. Initiate comparative studies examining how different NGRCs and HIS functionalities influence specific intervention effectiveness, which would further inform the design of evidence-based interventions.

**Generalizability**

1. Explore the unique challenges and context-specific solutions relevant to countries outside the United States.

### Question 2: Question Regarding NGRCs

The dominance of structured, measurable social needs interventions in the involved studies likely reflects that health care prioritizes short-term outcomes. In contrast, long-term lifestyle change interventions are overlooked. This may be due to operational challenges, including reimbursement constraints, lower adherence, long-term evaluation, etc [112-115]. However, lifestyle change interventions are crucial for preventing NCDs. To raise attention on lifestyle change interventions, incorporating NGRCs is beneficial for improving adherence and effectiveness. Future studies should focus on leveraging NGRC data to design and evaluate personalized, NGRC-focused lifestyle interventions aiming at advancing patient-centered and preventive care.

Current research has largely overlooked NGRCs beyond transportation barriers within the “destination accessibility” dimension of built environments. Neglecting other 4D elements, as well as social (eg, community crime, neighborhood safety, and socioeconomic data; included in 4 studies) and natural environments (specifically air pollution; included in 1 study), may limit our understanding of how specific NGRCs can inform behavioral interventions. Within the built environment domain, the exploration of “density,” “distance,” and “design” in the “5D” framework was limited, and “diversity” was not addressed. Future studies should systematically explore other less-studied NGRCs, such as social, natural, and other built NGRCs, to inform more holistic solutions of behavioral interventions using HISs.

**Figure 4.**
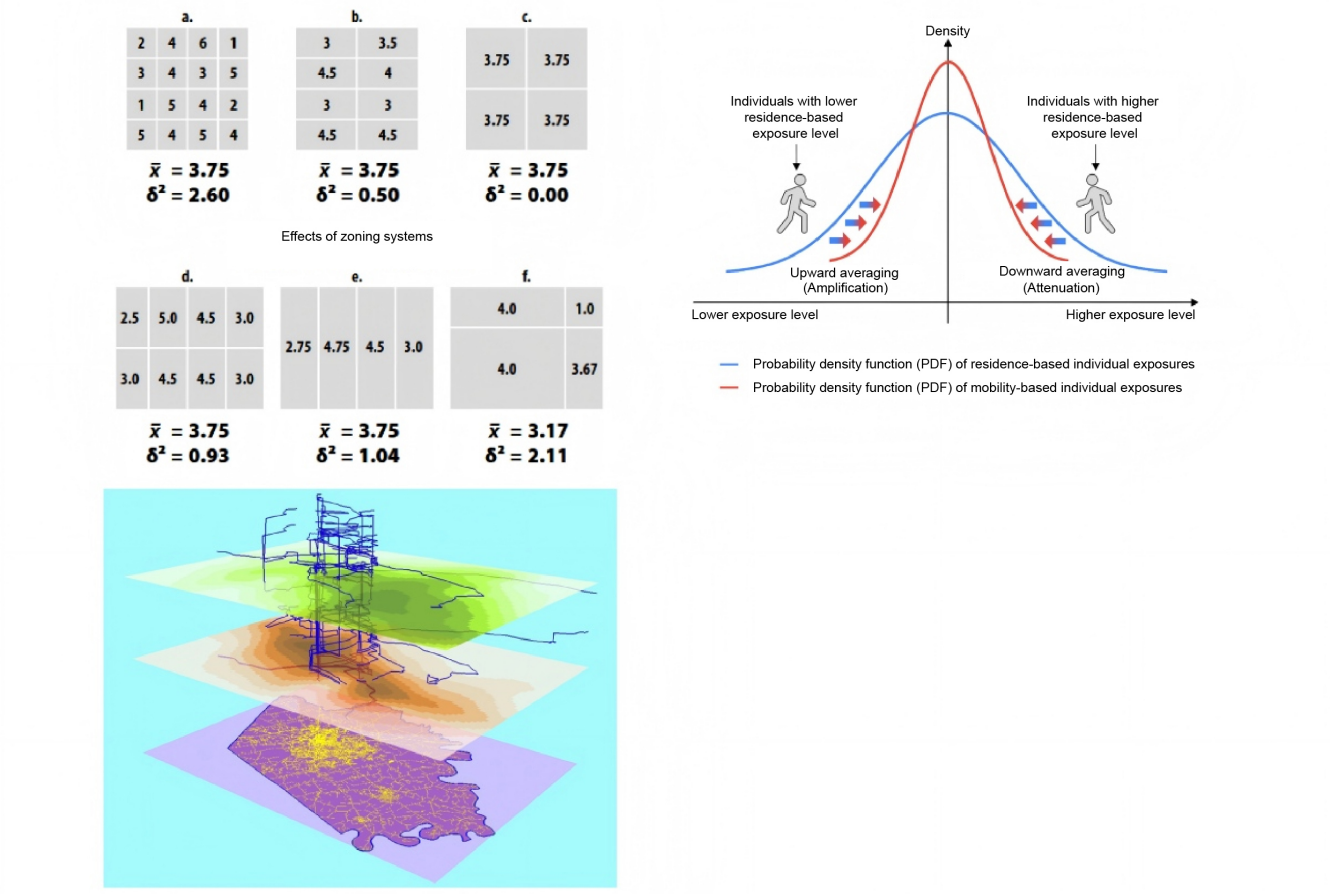
The top-left figure displays the modifiable areal unit problem. The mean value does not change with aggregation, but the variance declines [118,119]. The top-right figure presents how the neighborhood effect averaging problem operates in individual air pollution exposures [116,120]. The bottom figure shows the uncertain geographic context problem, which arises because of the spatial uncertainty in the actual areas that exert contextual influences on the individuals being studied, and the temporal uncertainty in the timing and duration in which individuals experienced these contextual influences [60,117].

Insufficient attention to objective, dynamic, and/or high-resolution NGRCs has left several key methodological challenges largely unaddressed. These include the modifiable areal unit problem, uncertain geographic context problem, neighborhood effect averaging problem, and problem of temporal variations in NGRCs (Figure 4) [60,116-120]. One likely reason is that 22 studies (92%) relied on actively collected, subjective, and static NGRC data, which require substantial participant effort and are therefore difficult to scale for capturing dynamic and/or high-resolution NGRCs. Passive data collection methods (eg, GPS tracking and linkage with public datasets) could help overcome these limitations by enabling more dynamic and/or high-resolution NGRC measurement. However, passive approaches alone cannot capture all real-time and/or high-resolution NGRCs, particularly those related to individual perceptions. Therefore, active data collection remains essential for assessing perceived NGRCs. While some studies have focused exclusively on perceived measures, others have underscored the added value of combining subjective and objective NGRCs to more comprehensively understand their associations with behavioral outcomes [121-124]. When collecting perceived dynamic and/or high-resolution NGRCs, issues, such as user vagueness and the quality of self-reported data, must be carefully considered. Future work should prioritize developing optimized and balanced approaches that integrate active and passive data collection, thereby leveraging dynamic and/or high-resolution NGRC measures to advance behavioral intervention research.

Furthermore, all involved studies illustrated the ongoing challenges of balancing granularity and comprehensiveness in assessing NGRCs. On the one hand, actively collected, subjective NGRC measures provide individualized insights but are limited in granularity and comprehensiveness. On the other hand, passively collected, objective indicators offer broad coverage but often lack individual insights and granularity, potentially due to existing dataset constraints. This trade-off underscores the need for integrated approaches that combine active and passive data to capture the complex, dynamic interplay between NGRCs and health behaviors.

To improve the granularity and comprehensiveness of NGRCs in various behavioral interventions, especially lifestyle change interventions, HISs—preferably tethered or interconnected PHRs in outpatient settings—should be considered. We have summarized 3 recommendations in Textbox 3.

### Question 3: Question Regarding Stakeholders’ Insights

Improved perceived value, convenience, patient autonomy, and privacy were consistently reported as outcomes of integrating technological (HISs) and content (NGRCs) innovations into behavioral interventions in all 10 included studies. On the other hand, social concerns persisted, including provider workload and the potential for discrimination. These benefits and concerns could be influenced by participants’ computer literacy, which was rarely reported in the studies. Given this gap, future research should explore how to balance technological and content innovations with unresolved social concerns, accounting for variations in computer literacy.

The NGRC information needs of providers, the clinical usefulness of NGRC data, and the usefulness of NGRC-related data visualizations are not yet well supported by existing evidence. Future studies should investigate which NGRCs should be presented in HISs, how they should be used in practice, and which interactive features best support their use.

Stakeholders’ insights into the integration of NGRCs and/or HISs into lifestyle interventions were not explored in the included studies. This highlights a critical gap in both technological and content innovations, as well as in addressing social concerns and in the integration of HISs and NGRCs into lifestyle change interventions.

We have summarized 5 recommendations focusing on enhancing NGRC and/or HIS integrations in behavioral interventions in Textbox 3.

### Question 4: Question Regarding Research Design

We found limited coverage and inconsistent health outcomes of NGRC-focused behavioral interventions using HISs. Health outcomes focused mainly on health care utilization (eg, emergency visits), with limited coverage of clinical indicators, including medication adherence [97], asthma control [92], cancer screening [98], and blood pressure [87]. This limited coverage of clinical health outcomes limits the understanding of long-term behavior change and highlights the need for more comprehensive assessments. Moreover, the inconsistent health care utilization outcomes [88,91] may be caused by the inconsistent and/or insufficient reporting of 5 aspects. These aspects include (1) NGRC attributes (eg, definitions and spatiotemporal characteristics), (2) HIS types and functionalities, (3) assessment methods (eg, clinical metrics [eg, HbA_1c_] and health care utilization), (4) stakeholders (eg, only HISs or various health providers), and (5) targeted diseases. Such heterogeneity causes low interstudy comparability and reproducibility. It also limits the ability to determine which behavioral interventions, leveraging specific NGRCs via particular HIS functions, are most effective, thereby complicating the translation of findings into evidence-based practice.

No studies have addressed key issues such as cost-effectiveness and ethical considerations. Future research should examine these critical factors to ensure successful implementation.

To develop evidence, we have summarized 3 recommendations in Textbox 3.

### Generalizability of the Results

Our objective was to synthesize global literature on NGRC-focused behavioral interventions supported by HISs. However, we were unable to identify published, peer-reviewed studies conducted outside of the United States. Initiatives, such as the European Health Data Space (EHDS) [125] and China’s Healthy China Initiative [126], reflect growing efforts to integrate SDoH, including NGRCs, into HISs. On one hand, contextual differences do not preclude transferability. The contextual differences include cultural norms, socioeconomic conditions, and geographic contexts. With appropriate localization and system-level adaptation, the core principles identified in United States–based studies can be effectively applied in diverse settings. However, the limited diversity of study settings may obscure unique challenges and solutions relevant to low-resource or non-Western countries. Addressing these gaps is essential, and future research should ensure that context-specific NGRC-focused behavioral interventions using HISs are inclusive, adaptable, and globally relevant (Table 3). On the other hand, technological and political differences across the world do not influence the generalizability of our study. These differences include (1) HIS infrastructure, architecture, and integration capabilities, and (2) data standards, privacy regulations (eg, the European General Data Protection Regulation [127]), and governance policies. The commonalities of these differences help support the generalizability of the findings [128].

### Strengths and Limitations

Our review has 4 key strengths. First, this article reviews the current state of NGRC-focused behavioral interventions using HISs. This provides a comprehensive analysis from 3 perspectives: technology, NGRC content, and stakeholders’ insights. Second, we searched a wide range of databases, leading to publications from diverse disciplines, including medical journals, environmental journals, and computer science conferences. Third, we consulted a library information specialist to develop the search strategy, ensuring its comprehensiveness and accuracy, which thus enhanced the research’s quality and credibility. Finally, to provide readers with sufficient context and allow them to assess the reliability of the evidence, we extracted and reported descriptive information, including study type, publication year, and sample size (Table S4 in Multimedia Appendix 1). We did not conduct a critical appraisal of the included studies, as it is not mandatory in scoping reviews. According to the Joanna Briggs Institute (JBI) methodology for scoping reviews [129], a critical appraisal is generally unnecessary unless it is directly relevant to the review objectives. Additionally, most quality assessment tools, including the Mixed Methods Appraisal Tool (MMAT) [130], primarily evaluate study design, data collection, and methodological rigor. Therefore, applying such tools would not provide meaningful information for our objectives (eg, HIS functionalities and NGRC indicators).

Our study also has several limitations that should be considered. First, NGRCs are ambiguous, suggesting that they are open to multiple interpretations or are vague. For example, in various studies, neighborhood safety may refer to different things, but there are no clear details of this concept in the studies. However, resolving this ambiguity is challenging because of the lack of standardized definitions and the context-dependent characteristics of NGRCs. Therefore, future studies should adopt detailed and consistent definitions of NGRCs, ideally guided by standardized terminologies or ontologies. These terminologies or ontologies can leverage and expand existing terminologies (eg, “community details” in Systematized Nomenclature of Medicine—Clinical Terms [SNOMED CT] and Logical Observation Identifiers Names and Codes [LOINC]) and SDoH ontologies [24].

### Possible Mechanisms and Implications for Clinicians or Policymakers

HISs are often seen as passive recorders of health data. However, tethered or interconnected PHRs can actively participate in the entire process of NGRC-focused behavioral interventions, especially in outpatient lifestyle change interventions, with the relatively limited help of health care providers. For example, platforms like the iManageCancer platform for cancer patients and the iManageMyHealth app for their physicians provide personalized content from reliable public resources on cancer. However, these platforms do not currently consider NGRC factors and focus on behavioral interventions [3]. This underscores the importance for providers and policymakers to utilize tethered or interconnected PHRs to collect comprehensive, dynamic, and static NGRCs; deliver timely behavioral interventions in outpatient settings; monitor behaviors and the health status; and assess interventions. By doing so, they can achieve public health goals and promote health equity.

### Conclusion

In this study, we found that most NGRC-focused behavioral interventions utilized transportation barriers to support social needs interventions. Meanwhile, HISs were involved only in specific aspects of the NGRC-focused behavioral intervention process. To achieve comprehensive dynamic and/or high-resolution NGRC considerations and the full integration of HISs throughout the behavioral intervention process, given the limited and heterogeneous evidence, further research is needed to determine how tethered or interconnected PHRs can effectively support NGRC-focused interventions, especially lifestyle change interventions. During implementation, it is essential to address social concerns alongside technological (eg, HIS integration) and content innovations (eg, NGRC integration). We compiled a list of 15 recommendations for future research. These recommendations are likely to accelerate the integration of tethered or interconnected PHRs in NGRC-focused behavioral interventions.

## Supplementary material

10.2196/77296 Multimedia Appendix 1 Additional information to support the scoping review.

10.2196/77296 Checklist 1 PRISMA-ScR checklist

## Abbreviations

A-CHESS: Addiction Comprehensive Health Enhancement Support System
EHR: electronic health record
EMR: electronic medical record
GRC: geo-referenced context
HIS: health information system
NCD: noncommunicable disease
NGRC: neighborhood geo-referenced context
PHR: personal health record
SDoH: social determinants of health
