# Supplementary material for: Using Health Information Systems to Support Behavioral Interventions in Local Contexts: Scoping Review
Source: JMIR Med Inform. 2026 Feb 25;14:e77296. doi: 10.2196/77296 (PMC12935427; doi:10.2196/77296)
Supplement: Multimedia Appendix 1 [file medinform-v14-e77296-s001.docx]

## Multimedia Appendix

This multimedia Appendix has six tables. These tables presents (1) related concepts, (2) search terms, (3) extracted information, (4) overview of characteristics of included studies, (5) summary of participants’ types, focused NCDs, health and computer literacy, and types of health settings and HISs for the included studies, and (6) the focal topic (NGRCs or HISs) and insights from various stakeholders regarding using HISs and/or NGRCs in behavioral interventions.

**Table S1.** Related concepts.

|  | Concepts | Attributes | Descriptions |
| --- | --- | --- | --- |
| **Health Information Systems (HIS)** | | | |
|  | EMRs (Hospital-based systems) | Used primarily by health providers to support decision-making in health care delivery. | Computerized health information systems that collect, store, and display patient information within a single hospital setting [1]. |
|  | EHRs (Hospital-based systems) |  | Securely stored and exchanged computerized health information systems, accessible across multiple healthcare settings [2]. |
|  | Standalone PHRs | Used by individuals to access, manage, and selectively share their health information with others for whom they are authorized, such as health providers, in a private, secure, and confidential environment [3]. | Entirely maintained and managed by patients or caregivers through software or mobile applications, and that are not sharable with healthcare institutions [4]. |
|  | Tethered PHRs (e.g., patient portals) |  | Computerized health information systems linked to a specific healthcare organization's electronic health record (EHR) system, with patients having limited access to information that the healthcare organization chooses to share. For example, a patient portal is a secure online website, managed by a health care organization, that provides patients access to their personal health information [5]. |
|  | Integrated PHRs |  | Computerized health information systems connected to a myriad of sources via application programming interfaces (APIs), encompassing healthcare providers, pharmacies, laboratories, and wearable devices, to provide a holistic perspective on an individual's health data. |
| **Neighborhood level** | | | |
|  | Neighborhood | 1. Static administrative areas (e.g., census tracts of their residential addresses); | |
|  |  | 2. Perceived neighborhood; | |
|  |  | 3. Various neighborhood delineations surrounding each participant's addresses (e.g., a 0.5-mile radius, a ten-minute walk, or a twenty-minute drive from the participant’s residential address); | |
|  |  | 4. Geographic areas encompassing all locations visited by an individual in their daily activities [6,7]. | |
| **Geo-referenced contexts (GRCs)** | | | |
|  | Built environments from a 5Ds perspective [8] | 1.Density: The variable of interest (e.g., dwelling unit) per area unit [8]; | |
|  |  | 2. Diversity: The arrangement and distribution of various land uses within a given area, based on land area, floor space, or employment [8,9]; | |
|  |  | 3. Design: The characteristics of the street network and facilities (e.g., walking environment quality [10,11], and neighborhood housing quality); | |
|  |  | 4. Distance (e.g., access to public transport [12]); | |
|  |  | 5. Destination accessibility: The assessments of the convenience of reaching (local or regional) attractors [8]. | |
|  | Natural environments | 1. Green and blue spaces (e.g., forests, mountains, and rivers) [13]; | |
|  |  | 2. Day-night cycle [14]; | |
|  |  | 3. Temperature, weather, and seasons [15–17]; | |
|  |  | 4. Air pollution and water quality [18,19]. | |
|  | Social environments | 1. Neighborhood economic and education conditions; | |
|  |  | 2. Neighborhood race/ethnic and age composition; | |
|  |  | 3. Neighborhood housing quality (e.g., median home value and median gross value); | |
|  |  | 4. Neighborhood social relationships (e.g., community support and neighborhood safety). | |
| **Social determinants of health (SDoHs)** | | | |
|  | Social determinants of health (SDoHs) | 1. Economic stability;  2. Education access and quality;  3. Health care access and quality;  4. Neighborhood and built environment;  5. Social and community context. | The conditions in which people are born, grow, live, work, and age, along with the wider set of forces and systems shaping the conditions of daily life [20]. |
|  | SDoH screening |  | SDoH screening comprises questions on topics such as education, NGRCs, and economic status. The results of these screenings can be utilized to assist in determining appropriate SDoH interventions. These screenings may be paper-based, verbal, or uploaded to electronic platforms (e.g., patient portals, EHRs, or digital platforms linked with HISs). |
| **Behavioral change interventions** | | | |
|  | Behavioral change interventions can be defined as coordinated sets of activities designed to change specified behavior patterns [21]. | | |
|  | Lifestyle change interventions | These interventions encourage people who are at high risk for a particular disease to do something about it [22]. | |
|  | Social needs interventions | Targeted at behaviors that improve or address a person’s SDoH (e.g., changing behaviors by modifying the transportation that supports them) [22]. | |
|  | Whole care | The entire healthcare process incorporates behavioral interventions as a key component. | |
| **Chronic care model (CCM)** | | | |
|  | | 1. Healthcare organization that has the expertise to provide appropriate clinical and behavioral management. | A useful framework for patient empowerment, self-management support, and improving clinical and behavioral outcomes [25–28]. |
|  |  | 2. Patient-oriented community resources through linkages with the relevant agencies; |  |
|  |  | 3. Patient-oriented self-management support in the knowledge, confidence, and skills for self-management of their condition; |  |
|  |  | 4. Delivery system design that encourages and enables productive interactions between patient-centered interdisciplinary teams; |  |
|  |  | 5. Provider-oriented decision support, providing the healthcare team with adequate knowledge of evidence-based guidelines for prevention; |  |
|  |  | 6. Clinical information systems to ensure timely, relevant data access about individual patients and populations of patients from clinical information systems for patients, caregivers, and health providers [23,24]. |  |
| **The role of HISs in the four stages of NGRC-focused behavioral interventions** | | | |
|  | NGRC data capture | 1. Collect NGRC data through embedded surveys in HISs; | |
|  |  | 2. Document NGRCs from other collection methods (e.g., paper) in HISs; | |
|  |  | 3. Link with external datasets (e.g., public health datasets) in HISs. | |
|  | Intervention prescription | Prescribed either automatically or by health providers. | These prescriptions are documented within HISs, ensuring that they are accessible for further monitoring and adjustment. |
|  | Monitoring | HISs track the progress and implementation of prescribed behavioral interventions over time. This includes monitoring adherence to interventions and recording relevant outcomes, such as changes in patient behavior or health metrics. | |
|  | Assessment | HIS data are used to evaluate the effectiveness of NGRC-focused interventions. Based on this assessment, interventions can be adjusted as needed to improve patient outcomes, ensuring that care remains personalized and responsive to the individual’s needs. | |

**Table S2.** Search terms.

| Search databases | | |
| --- | --- | --- |
| **Scopus** | | |
|  | (((TITLE-ABS-KEY((("electronic health" or personal or digital or medical or patient or participant) W/1 record) OR (("electronic health" or personal or digital or medical or patient or participant or subject or adult) W/2 data) OR ehr or referral OR ((screen or screening) W/4 ("social determinant" or "social need" or sdoh))) OR ABS("clinical data" or "clinical information" or "clinical record")) AND TITLE-ABS-KEY(((socio or socioeconomic or social or psychosocial) W/0 (risk or stress or vulnerable or vulnerability or disadvantage or status)) or (((socio or socioeconomic or social or psychosocial) W/1 (need or environment)) or "health need") or ((socio or socioeconomic or social or psychosocial) W/2 (determinant or exposure or factor or problem)) or sdoh or hrsn or health equity or patient contextual or "life circumstance" or (socioeconomic W/0 (factor or deprivation or status)) or (socio W/1 (factor or deprivation or status)) or (community and (prevalence or risk)) or (community W/1 (type or context or factor or health*)) or (environment* PRE/0 (factor or design)) or ((community or environment*) W/2 exposure)) AND (TITLE-ABS-KEY(("residence characteristic" or "place of birth" or "birth place" or domicile or "health service area" or "catchment area" or neighbourhood or housing or census or urban or rural or county or counties or household) OR geocode or geocoding or "geo-code" or "geo-coding" OR (geograph W/2 (code or coding or data or divers or distribute or distribution or disparity or inequity or information or measure or measurement or record or residence)) OR ((patient W/0 context) or (contextual W/1 data)) OR ((demograhic W/2 (data or information or record)) or census) OR ((area or community) W/2 (factor or resource or research or impact or level or link or linked or linking or linkage)) OR (social W/2 information)) or ABS((demograhic W/3 factor) OR ((regional or region) W/9 patient) OR ((personal or individual) W/1 (sdoh or "social determinant")) OR "family centered" OR (social W/2 (risk or problem or factor)) OR geography or geographies)) OR (TITLE-ABS-KEY(((socio or socioeconomic or social or psychosocial) W/0 (risk or stress or vulnerable or vulnerability or disadvantage or status)) or (((socio or socioeconomic or social or psychosocial) W/1 (need or environment)) or "health need") or ((socio or socioeconomic or social or psychosocial) W/2 (determinant or exposure or factor or problem)) or sdoh or hrsn or health equity or patient contextual or "life circumstance" or (socioeconomic W/0 (factor or deprivation or status)) or (socio W/1 (factor or deprivation or status)) or (community and (prevalence or risk)) or (community W/1 (type or context or factor or health*)) or (environment* PRE/0 (factor or design)) or ((community or environment*) W/2 exposure)) AND TITLE-ABS-KEY((community W/1 (register or registry or data or record or program or provider)) or "community health center" or "community mental health center"))) AND (TITLE-ABS-KEY(((health or unhealth) PRE/1 behavior) or ((healthy or unhealthy) PRE/1 behavior) or smoking or tobacco or diet or food or nutrition or lifestyle or alcohol or "physical activit" or sedentary or (("social determinant" or SDOH) W/0 (screen or screening))))) OR (( TITLE-ABS-KEY ( "chronic care model" ) ) AND ( TITLE-ABS-KEY ( ( "residence characteristic" OR "place of birth" OR "birth place" OR domicile OR "health service area" OR "catchment area" OR neighbourhood OR housing OR census OR urban OR rural OR county OR counties OR household ) OR geocode OR geocoding OR "geo-code" OR "geo-coding" OR ( geograph W/2 ( code OR coding OR data OR divers OR distribute OR distribution OR disparity OR inequity OR information OR measure OR measurement OR record OR residence ) ) OR ( ( patient W/0 context ) OR ( contextual W/1 data ) ) OR ( ( demograhic W/2 ( data OR information OR record ) ) OR census ) OR ( ( area OR community ) W/2 ( factor OR resource OR research OR impact OR level OR link OR linked OR linking OR linkage ) ) OR ( social W/2 information ) ) OR ABS ( ( demograhic W/3 factor ) OR ( ( regional OR region ) W/9 patient ) OR ( ( personal OR individual ) W/1 ( sdoh OR "social determinant" ) ) OR "family centered" OR ( social W/2 ( risk OR problem OR factor ) ) OR geography OR geographies ) ) AND ( TITLE-ABS-KEY ( ( ( socio OR socioeconomic OR social OR psychosocial ) W/0 ( risk OR stress OR vulnerable OR vulnerability OR disadvantage OR status ) ) OR ( ( ( socio OR socioeconomic OR social OR psychosocial ) W/1 ( need OR environment ) ) OR "health need" ) OR ( ( socio OR socioeconomic OR social OR psychosocial ) W/2 ( determinant OR exposure OR factor OR problem ) ) OR sdoh OR hrsn OR health AND equity OR patient AND contextual OR "life circumstance" OR ( socioeconomic W/0 ( factor OR deprivation OR status ) ) OR ( socio W/1 ( factor OR deprivation OR status ) ) OR ( community AND ( prevalence OR risk ) ) OR ( community W/1 ( type OR context OR factor OR health* ) ) OR ( environment* PRE/0 ( factor OR design ) ) OR ( ( community OR environment* ) W/2 exposure ) ) )) | |
| **Medline(Ovid)** | | |
|  | 1 | exp Medical Records Systems, Computerized/ or Health Records, Personal/ or "referral and consultation"/ |
|  | 2 | ((electronic health or personal or digital or medical or patient? or participant?) adj2 record?).ab,kf,ti. |
|  | 3 | ((electronic health or personal or digital or medical or patient? or participant? or subject? or adult?) adj3 data).ab,kf,ti. |
|  | 4 | (ehr or referral?).ab,kf,ti. |
|  | 5 | (clinical data or clinical information or clinical record?).ab. |
|  | 6 | (screen* adj5 (social determinant? or social need? or sdoh)).ab,ti. |
|  | 7 | or/1-6 [PHR] |
|  | 8 | social determinants of health/ or exp socioeconomic factors/ or health equity/ or environment design/ or residence characteristics/ |
|  | 9 | ((socio or socioeconomic* or social or psychosocial) adj (risk? or stress or vulnerab* or disadvantage? or status)).ab,hw,kf,ti. |
|  | 10 | (((socio or socioeconomic* or social or psychosocial) adj2 (need? or environment*)) or health needs).ab,kf,ti. |
|  | 11 | ((socio or socioeconomic* or social or psychosocial) adj3 (determinant? or exposure or factor? or problem?)).ab,kf,ti. |
|  | 12 | (sdoh or hrsn? or health equity or patient contextual or life circumstance?).ab,kf,ti. |
|  | 13 | (socioeconomic* adj (factor? or deprivation or status)).ab,kf,ti. |
|  | 14 | (socio adj2 (factor? or deprivation or status)).ab,kf,ti. |
|  | 15 | (communit* and (prevalence or risk?)).ab,kf,ti. |
|  | 16 | (communit* adj2 (type or context* or factor? or health)).ab,kf,ti. |
|  | 17 | (environment* adj (factor? or design)).ab,kf,ti. |
|  | 18 | ((communit* or environment*) adj3 exposure).ab,kf,ti. |
|  | 19 | or/8-18 [II social determinants of health] |
|  | 20 | residence characteristics/ or Catchment Area, Health/ or exp neighborhood characteristics/ or censuses/ or population health/ |
|  | 21 | (residence characteristic? or "place of birth" or birth place or domicile? or health service area? or catchment area? or neighbo?rhood? or housing or census* or urban or rural or county or counties or household?).ab,kf,ti. |
|  | 22 | (geocod* or geo-cod*).mp. |
|  | 23 | (geograph* adj3 (cod* or data or divers* or distribut* or disparit* or inequit* or information or measur* or record? or residen*)).ab,kf,ti. |
|  | 24 | (geography or geographies).ab. |
|  | 25 | ((patient? adj context) or (contextual adj2 data)).ab,kf,ti. |
|  | 26 | censuses/ or census tract/ |
|  | 27 | ((demograhic* adj3 (data or information or record?)) or census*).ab,kf,ti. |
|  | 28 | (demograhic* adj4 factor?).ab. |
|  | 29 | ((area or community) adj3 (factor? or resource? or research or impact* or level or link*)).ab,kf,ti. |
|  | 30 | ((regional or region) adj10 patient?).ab. |
|  | 31 | ((personal or individual) adj2 (sdoh or social determinant?)).ab. |
|  | 32 | family centered.ab. |
|  | 33 | (social adj3 (risk? or problem? or factor?)).ab. |
|  | 34 | (social adj3 information).ab,kf,ti. |
|  | 35 | or/20-34 [geographic context] |
|  | 36 | and/7,19,35 |
|  | 37 | community health centers/ or community mental health centers/ |
|  | 38 | ((community adj2 (regist* or data or record? or program? or provider?)) or community health center? or community mental health center?).ab,kf,ti. |
|  | 39 | 37 or 38 |
|  | 40 | 39 and 19 |
|  | 41 | 36 or 40 |
|  | 42 | health behavior/ or health risk behaviors/ or smoking cessation/ or smoking reduction/ or "tobacco use cessation"/ |
|  | 43 | ((health* or unhealth*) adj2 behavio?r*).mp. |
|  | 44 | (smoking or tobacco or diet or food or nutrition or lifestyle or alcohol or physical activit* or sedentary).mp. |
|  | 45 | ((social determinants or SDOH) adj screen*).ab,kf,ti. |
|  | 46 | or/42-45 [lifestyle] |
|  | 47 | 41 and 46 |
|  | 48 | chronic care model.ab,kf,ti. |
|  | 49 | and/48,19,35 |
|  | 50 | 47 OR 49 |

**Table S3.** Extracted information.

| 1: General information (including title, year, and country)  2: Population characteristics (type, number, gender, age, health literacy, computer literacy, and spoken language), disease, and health setting (e.g. primary, secondary, and tertiary care, as well as urban and rural settings)) | 3: Health information systems (HISs) | 4: Geo-referenced contextual attributes (GRC) | 5: Behavioral interventions | 6: Results  7: Conclusion and future studies. |
| --- | --- | --- | --- | --- |
|  | EMRs/EHRs/PHRs  Names  Short descriptions | N/B/S^a^  D/R/S/W^b^  Neighborhood types  GRC indicators  O/S^c^  Data sources(S/L/P^d^)  Capture frequency | L/S/E^e^  Interventions (Names, Prescriptions, Frequency, and Components (e.g., community resource referrals))  The relationship between HISs and behavioral interventions (C, DE, M, and A^f^)  Measurements of interventions |  |

a Built environment (B), Natural environments (N) or Social environments (S)

b Daily routes (D), Residential addresses (R), Work addresses (W), School addresses (S), Residential and work addresses (W and R), Individual addresses (I), and Zip code (Z)

C Objective (O), or Subjective (S)

d NGRC screenings (S), Location-aware devices (L), and Public datasets (P)

e Lifestyle change (individual) (L), social needs interventions (S), or entire care (E)

f Capture (C), Deliver (DE), Monitor (M), and Assess (A)

**Table S4.** Overview of characteristics of included studies.

| References | Study characteristics | | Participant characteristics | | | | | HISs (Type; Name) |
| --- | --- | --- | --- | --- | --- | --- | --- | --- |
|  | Year of publication | Study type | Types | Number | Males | Age | Language |  |
| [29] | 2019 | Quantitative descriptive | Patients | 1522 | 51.9% | Adults (mean age = 40) | English (76.9%), Haitian Cretole (8.2%), Portuguese (1.1%), Spanish (10.9%), Cape Verdean (3.1%) | EHR; Epic Systems Corporation (Epic) |
| [30] | 2023 | Before-and after quasi-experimental | Caregivers | 212 | - | - | English 45% | EHR; Epic |
| [31] | 2021 | Quantitative descriptive | Caregivers | 147 | 54.4% | Children (mean age = 11.4) | Only English | EHR; Epic |
| [32] | 2006 | RCT | Patients | 236 | 0% | Aged 40–64 years | - | Clinical information system |
| [33] | 2019 | Naturalistic | Caregivers | 198 | 0% | Aged 18 to 40 (mean age = 27.9) | - | EMR: The Kentucky River Community Care (KRCC) electronic medical record (EMR) |
| [34] | 2021 | Quantitative descriptive | Health providers | 26(including 5 general pediatricians, 6 specialists, and an internal medicine physician) | - | - | - | EHR: The Cerner Millennium EHR (Cerner Corporation, Kansas City, MO) |
| [35] | 2024 | Quantitative, descriptive and qualitative | Patients | 85 | 58.8% | Aged 10 to 101 (mean age = 63) | English (98.4%), Spanish (0.9%), Haitian Creole (0.5%), other (0.1%) | EHR; E-Clinical Works (ECW) |
|  |  |  | Health providers | - | - | - | - |  |
| [36] | 2020 | Quantitative descriptive | Patients | 252 | 42.5% | Ages 18-86 years (mean age = 53.8) | English or Spanish | PHR; A tablet-based electronic patient portal connected with Epic |
|  |  |  | Health providers | 27 | - | - | - |  |
| [37] | 2008 | RCT | Patients | 1223 | 1223 | Aged 52-79 years (mean age = 51.2) | English 56.9%, Spanish 26.7% | EHR |
| [38] | 2023 | Quantitative, descriptive and qualitative | Patients | 268 | 64.2% | Aged ≥ 18(mean age=55.9) | - | EHR |
| [39] | 2020 | Quantitative, descriptive and qualitative | Patients | 210 | - | - | - | EHR |
| [40] | 2023 | Quantitative, descriptive and qualitative | Patients | 75 | 60.3% | Aged from 22 to 84 (mean age = 62) | - | EMR |
|  |  |  | Health providers | 8 (including 8 nurses) | - | - | - |  |
| [41] | 2023 | Before-and after quasi-experimental | Patients | 1217 | 36.7% | Aged 18 years and older (mean age = 71.9) | - | EHR |
|  |  |  | Health providers | 5 (including 5 pharmacists） | - | - | - |  |
| [42] | 2023 | Before-and after quasi-experimental | Patients | 1220 | 46% | Aged 18–65 years old (mean age=51.56) | English 87.3%, Spanish 7.3%, other 5.4% | EMR |
| [43] | 2023 | Cohort | Patients | 1764 | 56.5% | Adults (mean age = 70) | - | EMR |
| [44] | 2021 | Before-and after quasi-experimental | Patients | 311 | 32.2% | Aged 18 years and older (mean age = 46) | Only English | EHR |
| [45] | 2020 | Qualitative | Health providers | 20(including physicians, advanced practice providers, pharmacists, nurses, and medical assistants) | 35% | Aged from 20 to 69 | - | PHR: A patient portal or a web-based consumer informatics application named Patientwisdom, tethered to Epic Systems Corporation |
| [46] | 2021 | Quantitative, descriptive and qualitative | Patients | 69. | 7% | Mean age=60.1 | - | EMR. |
| [47] | 2024 | Quantitative descriptive | Caregivers | 1473 | 52.7% | Aged 0 to 18 years (mean age = 3.6) | English 93.8%, Spanish 4.1%, other 0.8% | PHR: A population health application, Healthy Planet, and a patient portal connected with EpicCare EHR |
| [48] | 2023 | Quantitative descriptive | Patients | 2687 | 37.3% | Aged ≥18 years (mean age 62.9 ± 14.8 years) | English 98.9% | EMR |
| [49] | 2014 | Before-and after quasi-experimental | Patients | 322 | - | Adolescent (12 to 22 years) | - | EHR |
| [50] | 2023 | RCT | Caregivers | 540 | 5.7% | Caregivers of a child younger than 18 years | - | EMR |
| [51] | 2023 | Quantitative, descriptive and qualitative | Patients | 13460 | - | Adults | - | EHR |
|  |  |  | Health providers | Nurses, residents, and attending physicians | - | - | - |  |
| [52] | 2018 | RCT | Patients | 1200 | 47.5% | Aged 55–77 years old (mean age = 62.3) | - | EMR |

**Table S5.** Summary of participants' types, focused NCDs, and types of health settings and HISs for the included study.

| Descriptions | n(%) | References |
| --- | --- | --- |
| **The type of participants** | | |
| Only health providers | 2(8) | [34,45] |
| Only patients | 13(54) | [29,32,33,37–39,42–44,46,48,49,52] |
| Only the caregivers of patients | 4(17) | [30,31,47,50] |
| Health providers and patients | 5(21) | [35,36,40,41,51] |
| **NCDs** | | |
| Cardiovascular disease | 2(8) | [32,48] |
| Substance use disorders (SUD) | 2(8) | [33,48] |
| Diabetes | 3(13) | [42,43,48] |
| Systemic lupus erythematosus (SLE) | 1(4) | [46] |
| Asthma | 2(8) | [48,49] |
| Depression | 1(4) | [48] |
| Heart failure | 1(4) | [48] |
| Chronic obstructive pulmonary disease (COPD) | 1(4) | [48] |
| Cancer | 1(4) | [48] |
| Alzheimer’s | 1(4) | [48] |
| Exclude patients with acute illnesses, dementia, metastatic cancer, schizophrenia, or end-stage diseases | 1(4) | [52] |
| No specific details of NCDs | 16(75) | [29–31,34–41,44,45,47,50,51] |
| **Health and computer literacy** | | |
| Low health literacy | 1 | [47] |
| Low computer literacy | 2 | [32,33] |
| **The types of health settings** | | |
| **Country** | | |
| US | 24(100) | [29–52] |
| **Urban/rural health settings** | | |
| Urban | 14(42) | [29–31,34–37,44,46,47,49–52] |
| Urban and suburban | 1(4) | [38] |
| Rural | 2(8) | [33,43] |
| Urban and rural | 1(4) | [48] |
| No specific details | 6(25) | [32,39–42,45] |
| **Primary, secondary, or tertiary care** | | |
| Primary | 11(46) | [29,33,35–38,42–44,47,52] |
| Tertiary | 9(38) | [30,31,34,39,40,46,49–51] |
| Primary, secondary and tertiary | 3(13) | [41,45,48] |
| No specific details | 1(4) | [32] |
| **HISs** | | |
| EHRs | 12(50) | [29–31,34,35,38,39,41,44,49,51,52] |
| EMRs | 8(33) | [33,37,40,42,43,46,48,50] |
| PHRs | 3(13) | [36,45,47] |
| No specific details | 1(4) | [32] |

**Table S6.** The focal topic (NGRCs or HISs) and insights from various stakeholders regarding using HISs and/or NGRCs in behavioral interventions.

|  | NGRCs | | | | HISs | | |
| --- | --- | --- | --- | --- | --- | --- | --- |
|  | P/C/HP | The types of NGRCs^a^ | Insights of stakeholders | | P/C/HP^b^ | The types of HISs | Insights of stakeholders |
| **Provider workload** | | | | | | | |
| [34] | HP | B/S | Needs and advice:  Prioritized: socioeconomic and demographic data, followed by air quality and food desert status.  Excluded: air quality (limited quality/availability)  Criticized: air quality (clinical usefulness), and food desert visualizations (clinically challenging)  Suggested: simple indicator for food deserts  Recommended: add data on crime, vaccination, and homelessness. | | HP | EHR | Advantages: Ease of use. |
| [36] |  | B |  |  | HP | PHR | Advantages: Aligned with best practices; saving time;  Concerns about unmet patient expectations. |
| [35] |  | B |  |  | HP | EHR | Disadvantages: Increased workload. |
| [41] |  | B |  |  | HP | EHR | Advantages: Easy into integrate to their workflow. |
| [51] | HP | B | Advantages: A necessary part of the intake process to provide appropriate care;  Disadvantages: Slight discomfort asking NGRCs. |  | HP | EHR | Disadvantages: Time constraints. |
| [53] |  | B |  |  | HP | PHR | Advantages: Knowing the patient's needs, preferences, goals, and agenda before the visit as a significant driver for use;  Disadvantages: Difficulty in incorporating into the time-compressed visit and incompatibility with their workflow. |
| [40] | HP | B | Advantage: Facilitating the identification of additional needs beyond current practice; |  | HP | EMR | Advantages: Clear, easy to use, quick to complete, simple to incorporate into the workflow, and comfortable for patients to answer. |
| **Patient autonomy** | | | | | | | |
| [36] |  | B |  | | P | PHR | Advantages: Easy to use; confident to use; Quick to learn. |
| [38] | P | B | Advantages: Important and comfortable. | | P | EHR | Advantages: Convenient and fast. |
| [39] |  | B |  | | P | EHR | Advantages: Quick to complete and suitable for both self-completion and verbal administration. |
| [39] |  | B |  | | HP | EHR | Advantages: Preferring self-completion of the screening; |
| [51] | P | B | Advantages: Comfortable. | |  |  |  |
| **Privacy concern** | | | | | | | |
| [38] |  | B |  | | P | EHR | Advantages: Private, and allowed them to respond without feeling judged. |
| [39] | HP | B | Disadvantages: Uncomfortable with potentially stigmatizing questions; | | HP | EHR | Disadvantages: Addressing patient concerns uncovered during the process. |
| [41] | HP | B | Advantage: Normalizing topic for patient openness; addressing root causes of medication nonadherence. | |  |  |  |
| [50] | C | B | Disadvantages: Reported discrimination, with the most common experiences being less courtesy, less respect, and poorer service. | |  |  |  |

a Built environment (B), Natural environments (N), or Social environments (S); b Patients (P), Caregivers (C), or Health providers (HP).

### References

1. Boonstra A, Broekhuis M. Barriers to the acceptance of electronic medical records by physicians from systematic review to taxonomy and interventions. BMC Health Serv Res 2010 Dec;10(1):231. doi: 10.1186/1472-6963-10-231

2. Health informatics — Electronic health record — Definition, scope and context. International Organization for Standardization (ISO); 2005. Report No.: ISO/TR 20514:2005. Available from: https://ndls.org.cn/standard/detail/00535aa5aab7f6ad12ae4c1a8e2f8788

3. Kim J-W, Ryu B, Cho S, Heo E, Kim Y, Lee J, Jung SY, Yoo S. Impact of personal health records and wearables on health outcomes and patient response: Three-arm randomized controlled trial. JMIR Mhealth Uhealth 2019 Jan 4;7(1):e12070. doi: 10.2196/12070

4. Tang PC, Lee TH. Your doctor’s office or the internet? Two paths to personal health records. N Engl J Med 2009 Mar 26;360(13):1276–1278. doi: 10.1056/NEJMp0810264

5. Irizarry T, DeVito Dabbs A, Curran CR. Patient portals and patient engagement: A state of the science review. J Med Internet Res 2015 June 23;17(6):e148. doi: 10.2196/jmir.4255

6. Johnston R, Cybriwsky R. Spatial behavior: A geographic perspective. Urban Geography 1998 Aug;19(6):582–584. doi: 10.2747/0272-3638.19.6.582

7. Kwan M-P. The uncertain geographic context problem. Annals of the Association of American Geographers 2012 Sept;102(5):958–968. doi: 10.1080/00045608.2012.687349

8. Venerandi A, Mellen H, Romice O, Porta S. Walkability Indices—The State of the Art and Future Directions: A Systematic Review. Sustainability 2024 Aug 6;16(16):6730. doi: 10.3390/su16166730

9. Rodríguez DA, Evenson KR, Diez Roux AV, Brines SJ. Land use, residential density, and walking. American Journal of Preventive Medicine 2009 Nov;37(5):397–404. doi: 10.1016/j.amepre.2009.07.008

10. Giles-Corti B. Socioeconomic status differences in recreational physical activity levels and real and perceived access to a supportive physical environment. Preventive Medicine 2002 Dec;35(6):601–611. doi: 10.1006/pmed.2002.1115

11. Giles-Corti B, Donovan RJ. Relative influences of individual, social environmental, and physical environmental correlates of walking. Am J Public Health 2003 Sept;93(9):1583–1589. doi: 10.2105/AJPH.93.9.1583

12. Crawford D, Cleland V, Timperio A, Salmon J, Andrianopoulos N, Roberts R, Giles-Corti B, Baur L, Ball K. The longitudinal influence of home and neighbourhood environments on children’s body mass index and physical activity over 5 years: The CLAN study. Int J Obes 2010 July;34(7):1177–1187. doi: 10.1038/ijo.2010.57

13. Zhang R, Wulff H, Duan Y, Wagner P. Associations between the physical environment and park-based physical activity: A systematic review. Journal of Sport and Health Science 2019 Sept;8(5):412–421. doi: 10.1016/j.jshs.2018.11.002

14. Luik AI, Zuurbier LA, Hofman A, Van Someren EJW, Tiemeier H. Stability and fragmentation of the activity rhythm across the sleep-wake cycle: The importance of age, lifestyle, and mental health. Chronobiology International 2013 Dec;30(10):1223–1230. doi: 10.3109/07420528.2013.813528

15. Feinglass J, Lee J, Semanik P, Song J, Dunlop D, Chang R. The effects of daily weather on accelerometer-measured physical activity. Journal of Physical Activity and Health 2011 Sept;8(7):934–943. doi: 10.1123/jpah.8.7.934

16. Ferguson T, Curtis R, Fraysse F, Olds T, Dumuid D, Brown W, Esterman A, Maher C. Weather associations with physical activity, sedentary behaviour and sleep patterns of australian adults: A longitudinal study with implications for climate change. Int J Behav Nutr Phys Act 2023 Mar 14;20(1):30. doi: 10.1186/s12966-023-01414-4

17. Kamiński M, Kręgielska-Narożna M, Bogdański P. Seasonal variation in lifestyle behavior in poland: Google searches and market sales analysis. BMC Public Health 2021 Dec;21(1):1516. doi: 10.1186/s12889-021-11543-9

18. Hu X, Knibbs LD, Zhou Y, Ou Y, Dong G-H, Dong H. The role of lifestyle in the association between long-term ambient air pollution exposure and cardiovascular disease: A national cohort study in China. BMC Med 2024 Mar 5;22(1):93. doi: 10.1186/s12916-024-03316-z

19. Strak M, Janssen N, Beelen R, Schmitz O, Karssenberg D, Houthuijs D, Van Den Brink C, Dijst M, Brunekreef B, Hoek G. Associations between lifestyle and air pollution exposure: Potential for confounding in large administrative data cohorts. Environmental Research 2017 July;156:364–373. doi: 10.1016/j.envres.2017.03.050

20. Social determinants of health - healthy people 2030 | odphp.health.gov. Available from: https://odphp.health.gov/healthypeople/priority-areas/social-determinants-health [accessed Oct 15, 2025]

21. Michie S, Van Stralen MM, West R. The behaviour change wheel: A new method for characterising and designing behaviour change interventions. Implementation Sci 2011 Dec;6(1):42. doi: 10.1186/1748-5908-6-42

22. National Research Council (US) Panel on Race, Ethnicity, and Health in Later Life. Critical perspectives on racial and ethnic differences in health in late life. Anderson NB, Bulatao RA, Cohen B, editors. Washington (DC): National Academies Press (US); 2004. PMID:20669464ISBN:978-0-309-09211-1

23. Nair BR, Browne W. Chronic disease management: Seizing the moment in medical education. Pharm Med 2008 Nov;22(6):351–354. doi: 10.1007/BF03256731

24. Wagner EH, Austin BT, Davis C, Hindmarsh M, Schaefer J, Bonomi A. Improving chronic illness care: Translating evidence into action. Health Affairs 2001 Nov;20(6):64–78. doi: 10.1377/hlthaff.20.6.64

25. Coleman K, Austin BT, Brach C, Wagner EH. Evidence on the chronic care model in the new millennium. Health Affairs 2009 Jan;28(1):75–85. doi: 10.1377/hlthaff.28.1.75

26. Siminerio LM, Piatt GA, Emerson S, Ruppert K, Saul M, Solano F, Stewart A, Zgibor JC. Deploying the chronic care model to implement and sustain diabetes self-management training programs. Diabetes Educ 2006 Mar;32(2):253–260. doi: 10.1177/0145721706287156

27. Siminerio LM, Piatt G, Zgibor JC. Implementing the chronic care model for improvements in diabetes care and education in a rural primary care practice. Diabetes Educ 2005 Mar;31(2):225–234. doi: 10.1177/0145721705275325

28. Stellefson M, Dipnarine K, Stopka C. The chronic care model and diabetes management in US primary care settings: A systematic review. Prev Chronic Dis 2013 Feb 21;10:120180. doi: 10.5888/pcd10.120180

29. Buitron De La Vega P, Losi S, Sprague Martinez L, Bovell-Ammon A, Garg A, James T, Ewen AM, Stack M, DeCarvalho H, Sandel M, Mishuris RG, Deych S, Pelletier P, Kressin NR. Implementing an EHR-based screening and referral system to address social determinants of health in primary care. Medical Care 2019 June;57(Suppl 2):S133–S139. doi: 10.1097/MLR.0000000000001029

30. Cordova-Ramos EG, Jain C, Torrice V, McGean M, Buitron De La Vega P, Burke J, Stickney D, Vinci RJ, Drainoni M-L, Parker MG. Implementing social risk screening and referral to resources in the NICU. Pediatrics 2023 Apr 1;151(4):e2022058975. doi: 10.1542/peds.2022-058975

31. Fortin K, Vasan A, Wilson-Hall CL, Brooks E, Rubin D, Scribano PV. Using quality improvement and technology to improve social supports for hospitalized children. Hospital Pediatrics 2021 Oct 1;11(10):1120–1129. doi: 10.1542/hpeds.2020-005800

32. Jilcott SB, Keyserling TC, Samuel-Hodge CD, Rosamond W, Garcia B, Will JC, Farris RP, Ammerman AS. Linking clinical care to community resources for cardiovascular disease prevention: The north carolina enhanced WISEWOMAN project. Journal of Women’s Health 2006 June;15(5):569–583. doi: 10.1089/jwh.2006.15.569

33. Johnston DC, Mathews WD, Maus A, Gustafson DH. Using smartphones to improve treatment retention among impoverished substance-using appalachian women: A naturalistic study. Subst�Abuse 2019 Jan;13:1178221819861377. doi: 10.1177/1178221819861377

34. Kane NJ, Wang X, Gerkovich MM, Breitkreutz M, Rivera B, Kunchithapatham H, Hoffman MA. The envirome web service: Patient context at the point of care. Journal of Biomedical Informatics 2021 July;119:103817. doi: 10.1016/j.jbi.2021.103817

35. Lynch S, Street NW, Sharpe L, Kellish A. Social determinants of health screening: Primary care PRAPARE tool implementation. The Journal for Nurse Practitioners 2024 Apr;20(4):104955. doi: 10.1016/j.nurpra.2024.104955

36. Palakshappa D, Benefield AJ, Furgurson KF, Harley MG, Bundy R, Moses A, Taxter AJ, Bensinger AS, Cao X, Denizard-Thompson N, Rosenthal GE, Miller DP. Feasibility of mobile technology to identify and address patients’ unmet social needs in a primary care clinic. Population Health Management 2021 June 1;24(3):385–392. doi: 10.1089/pop.2020.0059

37. Percac-Lima S, Grant RW, Green AR, Ashburner JM, Gamba G, Oo S, Richter JM, Atlas SJ. A culturally tailored navigator program for colorectal cancer screening in a community health center: A randomized, controlled trial. J GEN INTERN MED 2009 Feb;24(2):211–217. doi: 10.1007/s11606-008-0864-x

38. Russell LE, Cohen AJ, Chrzas S, Halladay CW, Kennedy MA, Mitchell K, Moy E, Lehmann LS. Implementing a social needs screening and referral program among veterans: Assessing circumstances & offering resources for needs (ACORN). J GEN INTERN MED 2023 Oct;38(13):2906–2913. doi: 10.1007/s11606-023-08181-9

39. Wallace AS, Luther B, Guo J-W, Wang C-Y, Sisler S, Wong B. Implementing a social determinants screening and referral infrastructure during routine emergency department visits, utah, 2017–2018. Prev Chronic Dis 2020 June 18;17:190339. doi: 10.5888/pcd17.190339

40. Wright QC, Wiencek CA, Williams IC. Utilizing a tailored approach to standardized screening of health-related social needs. Journal of Nursing Care Quality 2024 Apr;39(2):114–120. doi: 10.1097/NCQ.0000000000000745

41. Wu L, Chang C, Lo K, Butler K, Uratsu C, McCloskey J, Ranatunga D, Grant R, Deguzman L. Telephone-based social health screening by pharmacists in the nonadherent medicare population. JMCP 2023 Nov;29(11):1184–1192. doi: 10.18553/jmcp.2023.29.11.1184

42. Roth SE, Gronowski B, Jones KG, Smith RA, Smith SK, Vartanian KB, Wright BJ. Evaluation of an integrated intervention to address clinical care and social needs among patients with type 2 diabetes. J GEN INTERN MED 2023 Mar;38(S1):38–44. doi: 10.1007/s11606-022-07920-8

43. Tanumihardjo JP, Kuther S, Wan W, Gunter KE, McGrath K, O’Neal Y, Wilkinson C, Zhu M, Packer C, Petersen V, Chin MH. New frontiers in diabetes care: Quality improvement study of a population health team in rural critical access hospitals. J GEN INTERN MED 2023 Mar;38(S1):56–64. doi: 10.1007/s11606-022-07928-0

44. Bechtel N, Jones A, Kue J, Ford JL. Evaluation of the core 5 social determinants of health screening tool. Public Health Nursing 2022 Mar;39(2):438–445. doi: 10.1111/phn.12983

45. Holt JM, Cusatis R, Asan O, Williams J, Nukuna S, Flynn KE, Moore J, Crotty BH. Incorporating patient-generated contextual data into care: Clinician perspectives using the consolidated framework for implementation science. Healthcare 2020 Mar;8(1):100369. doi: 10.1016/j.hjdsi.2019.100369

46. Taber KA, Williams JN, Huang W, McLaughlin K, Vogeli C, Cunningham R, Wichmann L, Feldman CH. Use of an integrated care management program to uncover and address social determinants of health for individuals with lupus. ACR Open Rheumatology 2021 May;3(5):305–311. doi: 10.1002/acr2.11236

47. Stark K, Mathur M, Fok C, Le Y-C, Hunt ET, McCoy J, Mansoori S, Ukoh N, Keatts S, Fanous E, Eisenhauer R, McKay S. Evaluation of a clinic-based, electronic social determinants of health screening and intervention in primary care pediatrics. Academic Pediatrics 2024 Mar;24(2):302–308. doi: 10.1016/j.acap.2023.12.010

48. Gupta D, Self S, Thomas D, Supra J, Rudisill C. Understanding the role of a technology and EMR-based social determinants of health screening tool and community-based resource connections in health care resource utilization. Medical Care 2023 July;61(7):423–430. doi: 10.1097/MLR.0000000000001800

49. Britto MT, Vockell A-LB, Munafo JK, Schoettker PJ, Wimberg JA, Pruett R, Yi MS, Byczkowski TL. Improving outcomes for underserved adolescents with asthma. Pediatrics 2014 Feb 1;133(2):e418–e427. doi: 10.1542/peds.2013-0684

50. Glasser NJ, Lindau ST, Wroblewski K, Abramsohn EM, Burnet DL, Fuller CM, Miller DC, O’Malley CA, Shiu E, Waxman E, Makelarski JA, CommunityRx-Hunger Collaborators, Carter A, Ciaccio CE, Chase E, Darlington WS, DeAlmeida K, Jerome JS, Ott J, Verma R, Wang E. Effect of a social care intervention on health care experiences of caregivers of hospitalized children: A randomized clinical trial. JAMA Pediatr 2023 Dec 1;177(12):1266. doi: 10.1001/jamapediatrics.2023.4596

51. Loo S, Anderson E, Lin JG, Smith P, Murray GF, Hong H, Jacquet GA, Koul R, Rosenmoss S, James T, Shankar KN, De La Vega PB. Evaluating a social risk screening and referral program in an urban safety‐net hospital emergency department. JACEP Open 2023 Feb;4(1):e12883. doi: 10.1002/emp2.12883

52. Percac‐Lima S, Ashburner JM, Rigotti NA, Park ER, Chang Y, Kuchukhidze S, Atlas SJ. Patient navigation for lung cancer screening among current smokers in community health centers a randomized controlled trial. Cancer Medicine 2018 Mar;7(3):894–902. doi: 10.1002/cam4.1297

53. Holt JM, Cusatis R, Asan O, Williams J, Nukuna S, Flynn KE, Moore J, Crotty BH. Incorporating patient-generated contextual data into care: Clinician perspectives using the consolidated framework for implementation science. Healthcare 2020 Mar;8(1):100369. doi: 10.1016/j.hjdsi.2019.100369
